# Oral supplementation of nicotinamide riboside alters intestinal microbial composition in rats and mice, but not humans

**DOI:** 10.1038/s41514-023-00106-4

**Published:** 2023-04-03

**Authors:** A. Augusto Peluso, Agnete T. Lundgaard, Parizad Babaei, Felippe Mousovich-Neto, Andréa L. Rocha, Mads V. Damgaard, Emilie G. Bak, Thiyagarajan Gnanasekaran, Ole L. Dollerup, Samuel A. J. Trammell, Thomas S. Nielsen, Timo Kern, Caroline B. Abild, Karolina Sulek, Tao Ma, Zach Gerhart-Hines, Matthew P. Gillum, Manimozhiyan Arumugam, Cathrine Ørskov, Douglas McCloskey, Niels Jessen, Markus J. Herrgård, Marcelo A. S. Mori, Jonas T. Treebak

**Affiliations:** 1 Novo Nordisk Foundation Center for Basic Metabolic Research, Faculty of Health and Medical Sciences, University of Copenhagen, Copenhagen, Denmark; 2 Novo Nordisk Foundation Center for Biosustainability, Technical University of Denmark, Kgs. Lyngby, Denmark; 3 Department of Biochemistry and Tissue Biology, Institute of Biology, University of Campinas, Campinas, Brazil; 4 Steno Diabetes Center Aarhus, Aarhus University Hospital, Aarhus, Denmark; 5 Department of Biomedical Sciences, Faculty of Health and Medical Sciences, University of Copenhagen, Copenhagen, Denmark; 6 Department of Clinical Medicine, Aarhus University, Aarhus, Denmark; 7 Steno Diabetes Center Copenhagen, Herlev Hospital, Herlev, Denmark; 8 Department of Biomedicine, Aarhus University, Aarhus, Denmark; 9 BioInnovation Institute, Copenhagen, Denmark; 10 Obesity and Comorbidities Research Center, University of Campinas, Campinas, SP Brazil; 11 Experimental Medicine Research Cluster, University of Campinas, Campinas, SP Brazil

**Keywords:** Microbiology, Obesity, Obesity, Rat, Translational research

## Abstract

The gut microbiota impacts systemic levels of multiple metabolites including NAD^+^ precursors through diverse pathways. Nicotinamide riboside (NR) is an NAD^+^ precursor capable of regulating mammalian cellular metabolism. Some bacterial families express the NR-specific transporter, *PnuC*. We hypothesized that dietary NR supplementation would modify the gut microbiota across intestinal sections. We determined the effects of 12 weeks of NR supplementation on the microbiota composition of intestinal segments of high-fat diet-fed (HFD) rats. We also explored the effects of 12 weeks of NR supplementation on the gut microbiota in humans and mice. In rats, NR reduced fat mass and tended to decrease body weight. Interestingly, NR increased fat and energy absorption but only in HFD-fed rats. Moreover, 16S rRNA gene sequencing analysis of intestinal and fecal samples revealed an increased abundance of species within *Erysipelotrichaceae* and *Ruminococcaceae* families in response to NR. *PnuC*-positive bacterial strains within these families showed an increased growth rate when supplemented with NR. The abundance of species within the *Lachnospiraceae* family decreased in response to HFD irrespective of NR. Alpha and beta diversity and bacterial composition of the human fecal microbiota were unaltered by NR, but in mice, the fecal abundance of species within *Lachnospiraceae* increased while abundances of *Parasutterella* and *Bacteroides dorei* species decreased in response to NR. In conclusion, oral NR altered the gut microbiota in rats and mice, but not in humans. In addition, NR attenuated body fat mass gain in rats, and increased fat and energy absorption in the HFD context.

## Introduction

The increase in obesity and obesity-related diseases, such as diabetes and coronary heart disease, poses a growing challenge to healthcare systems worldwide^1^. Novel low-cost, non-invasive therapies are therefore needed. In preclinical animal models, nicotinamide riboside (NR) has emerged as a potential candidate to attenuate obesity and related metabolic diseases^2^.

NR is a naturally occurring vitamin B_3_ found in certain foods, most notably cow’s milk^3,4^ and yeast^5^. Like other B_3_ vitamins [e.g., nicotinamide (NAM) and nicotinic acid (NA)], NR is a nicotinamide adenine dinucleotide (NAD^+^) precursor that potently increases NAD^+^ levels in various cells and tissues^6^. NAD^+^ is an essential co-factor that is continuously reduced and oxidized in catabolic reactions of carbohydrates and fats to produce ATP, while its phosphorylated form (NADP^+^) is related to anabolic reactions, such as the synthesis of fatty acids and cholesterol^7^. Moreover, NAD^+^ is a co-substrate in reactions catalyzed by poly (ADP-ribose) polymerases (PARPs) and sirtuins (SIRTs) that regulate cellular pathways related to mitochondrial function, inflammation, and DNA repair^8^.

Obese individuals have decreased NAD^+^ levels in multiple tissues, and it has been speculated that nutrition-mediated increases in NAD^+^ levels can alleviate obesity and other metabolic diseases^9^. Only a few clinical studies have been conducted with orally supplemented NR^6,10–19^. These studies are generally conducted over shorter periods (i.e., weeks), and they show limited effects on metabolic parameters in overweight, middle-aged individuals, who constitute a substantial risk group for metabolic diseases. In contrast, NR supplementation improves several metabolic outcomes in mouse models, such as body weight, glucose tolerance, and serum cholesterol levels^8,9,20^. This apparent lack of translatability between species calls for additional preclinical studies where interspecies comparisons are made. Such investigations could uncover how conserved the various mechanisms of action of NR are and thereby shape more targeted future human interventions for potential therapeutic exploitation.

The gut microbiome contributes to the host metabolism through the production of molecules such as short-chain fatty acids (SCFAs) and vitamins by extracting energy from otherwise indigestible food sources (e.g., complex carbohydrates and fibers)^21–23^. The gut microbiome also plays an essential role in health and disease^24^. In disease states, the gut microbiome shifts from homeostasis to dysbiosis, and this is linked to a broad spectrum of conditions, including obesity^21^, atherosclerosis^25^, ulcerative colitis^26^, and colorectal cancer^27^. Thus, the gut microbiome is recognized as a potential therapeutic target to alleviate metabolic diseases^28^.

Diet has an essential role in shaping and changing the gut microbiome composition from neonatal to adult life^29,30^. For example, a diet rich in protein and fat and low in fibers decreases the bacterial species richness and diversity of the human gut microbiota^31–34^. Moreover, high-fat diet (HFD) feeding alters the relative abundances of certain bacterial species in the gut microbiota of mice^35–37^. Thus, diet has the potential to change the gut microbiome composition of the host, which, in part, may affect the overall metabolic health of the individual.

Notably, up to 40% of the bacterial species found in the human gut cannot synthesize NAD^+^ de novo and consequently depend on scavenging the environmentally available NAD^+^ precursors (e.g., NAM, NA, and NR) to sustain basic metabolic functions^38,39^. The genomic basis for NAD^+^ synthesis is largely phylum-specific, with *Bacteroidetes* and *Fusobacteria* being primarily de novo NAD^+^ producers, while a significant fraction of *Firmicutes* and *Actinobacteria* harbors NAD^+^ salvaging pathway genes^38^. Moreover, *PnuC* is an NR transporter found in some *Firmicutes* and *Proteobacteria* species, suggesting that NR serves as an NAD^+^ precursor for bacteria belonging to these phyla^38,40–43^. Consequently, oral NR supplementation could provide a selective advantage for NAD^+^ precursor-dependent species. In addition, the uptake and metabolism of NR by certain bacterial species could change the availability of NAD^+^ precursors accessible to the host^44^. This is particularly relevant as very little NR appears to reach target tissues intact^44,45^.

This study aimed to characterize the effects of NR supplementation on metabolic and microbial outcomes linked to obesity and metabolic syndrome. To this end, we evaluated the metabolic health as well as the microbiota composition of rats subjected to oral NR supplementation in combination with a low- or high-fat diet. Moreover, because bacterial communities and consequent physiological functions can vary in different parts of the intestinal tract^46,47^, we also examined the role of NR treatment on the fecal microbiota composition and lumen content from multiple intestinal sections. Finally, we extended the analysis of the gut microbiota composition to mice and humans treated orally with NR.

## Results

### NR supplementation reduces HFD-induced fat mass gain and tended to decrease body weight but not whole-body energy metabolism in rats

**Fig. 1:**
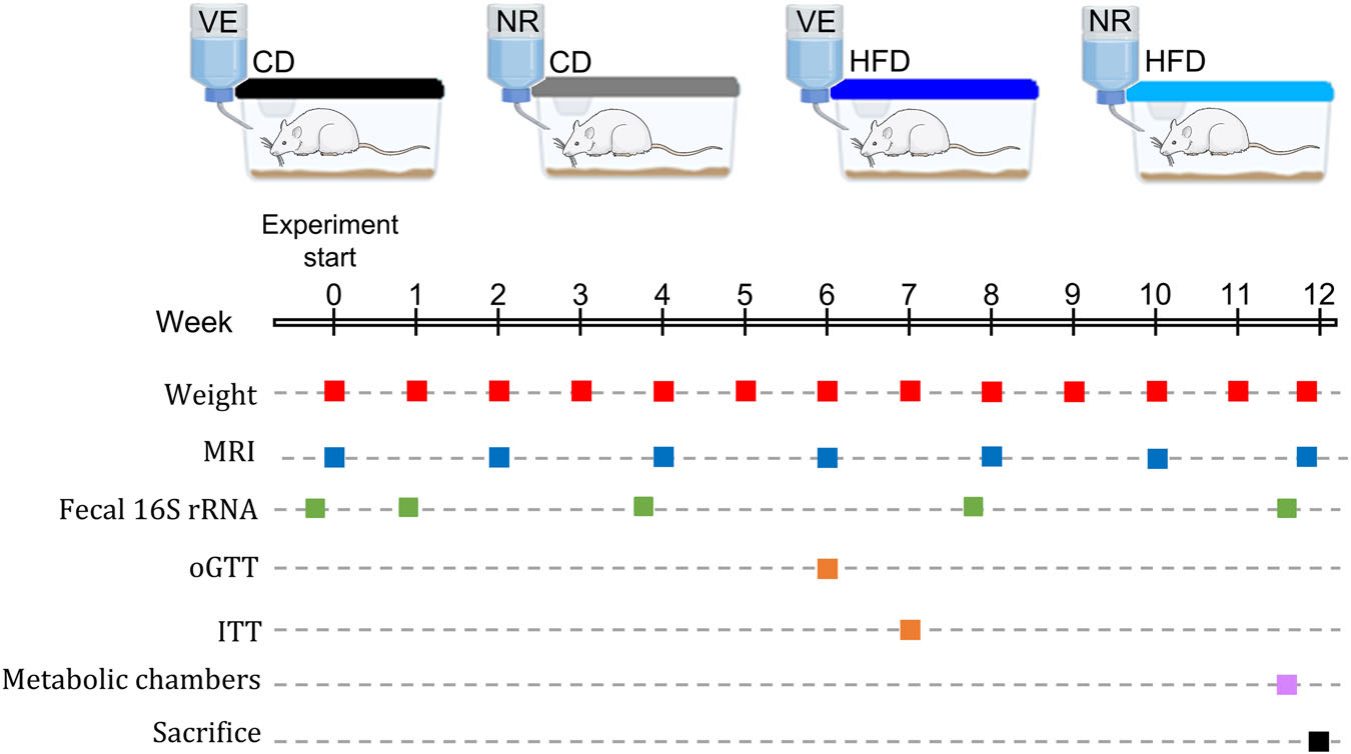
Temporal diagram design for physiological and metabolic experiments in rats. Rats were divided into four groups (*n* = 8) based on body weight upon arrival and received a standard chow diet and water. During the 12-week study period, they were fed an experimental 60% high-fat diet (HFD) or a matched control diet (CD) with 10% fat ad libitum in combination with 300 mg/kg/day nicotinamide riboside (NR) or vehicle (water, VE). During this period, they were subjected to an oral glucose tolerance test (oGTT, week 6) and an insulin tolerance test (ITT, week 7). In addition, fecal samples were collected at 5 time points, intestinal content from distinct sites was taken for 16S rRNA gene sequencing, body weight gain was monitored weekly, body composition was measured every second week and gas exchange, as well as feed intake, was assessed using metabolic chambers.

**Fig. 2:**
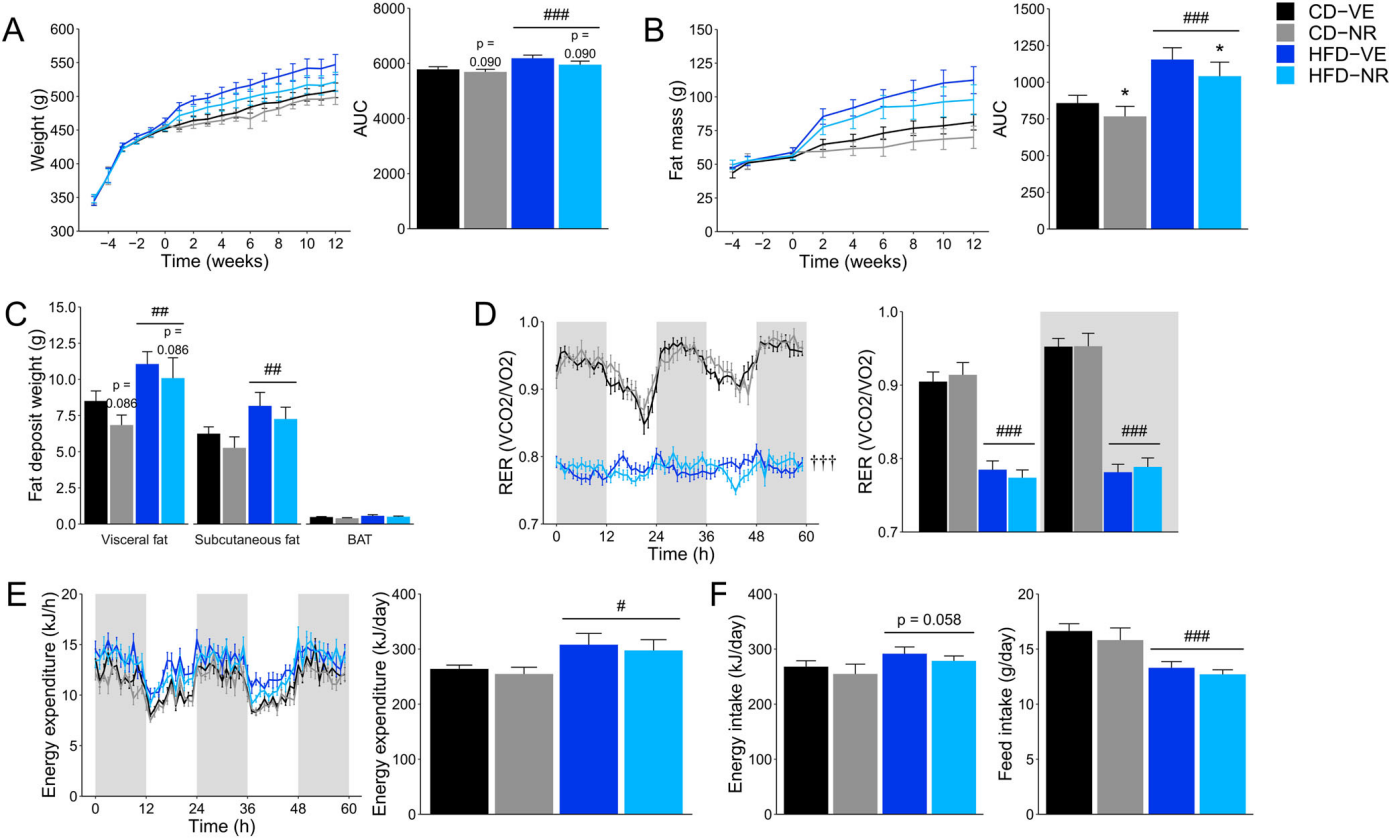
Rat whole-body phenotype characterization. **A** Body weight curve for animals over the 12 weeks study period (study start) with 4-5 weeks of acclimatization and average body weight gain over the 12 weeks. Diet effect: ###*p* < 0.001. **B** Fat mass curve for animals over the 12 weeks study period (study start) with a 3–4-week acclimatization period and the average fat mass gain average over 12 weeks. Diet effect: ###*p* < 0.001. NR effect: *p < 0.05. **C** Weight of fat deposits taken at sacrifice. Diet effect: ##*p* < 0.01. **D** Respiratory exchange ratio (RER). To the left, the measured RER over 60 h is depicted. To the right, the average RER for the dark and light phases is presented. Shading grey indicates dark phase periods. Diet effect: ###*p* < 0.001. Four-hour phase difference: †††*p* < 0.001. **E** Energy expenditure over 60 h in week 12 as well as daily average. Shading grey indicates dark phase periods. Diet effect: #*p* < 0.05. **F** Energy and feed intake in week 12. Diet effect: ###*p* < 0.001. Data are shown as mean ± SEM. *n* = 6–8.

The experimental protocol is shown in Fig. 1. Rats were fed a HFD or a matched control diet for 12 weeks and sampled repeatedly. As expected, the HFD increased body weight and fat mass (Fig. 2A, B). The increase in overall fat mass was attenuated by 300 mg/kg/day of NR supplementation, but it only tended to reduce body weight independently of diet (Fig. 2A). Moreover, NR tended to reduce visceral fat mass (Fig. 2C) and lean body mass (Supplementary Fig. 1), while we found no differences for subcutaneous and brown fat mass (Fig. 2C). To assess whole-body metabolism, animals were placed in metabolic chambers. Although NR did not affect energy substrate utilization based on the respiratory exchange ratio (RER), HFD-fed animals showed an expected reduced RER compared to the control diet (CD)-fed animals during both dark and light phases (Fig. 2D). In addition, while energy expenditure was higher and energy intake tended to be increased, food intake was lower in the HFD-fed groups. NR treatment did not affect these responses (Fig. 2E, F). We observed significant rhythmicity over 24 h in RER, energy expenditure, and food intake. Moreover, we found a significant 4-hour phase difference in RER rhythmicity between NR treatment groups in HFD-fed animals (Fig. 2D), while there were no differences in the rhythm of food intake or energy expenditure in the HFD groups and no difference in rhythm in any of the data sets between CD groups.

We performed oral glucose tolerance tests (OGTT), insulin tolerance tests (ITT) and quantified liver glycogen to determine potential changes in glucose metabolism. Glucose and insulin tolerances were impaired in the HFD-fed groups (Supplementary Fig. 2a–c) while glycogen levels did not change between the groups (Supplementary Fig. 2d). Moreover, we found no differences in circulating insulin levels or calculated HOMA-IR (Supplementary Fig. 2e), and NR supplementation did not affect any of the measured parameters. Collectively, while the HFD was efficient at promoting fat gain and impairing whole-body metabolism of rats, NR treatment reduced fat mass gain and affected the rhythmicity of RER but had no other significant metabolic effects in this metabolic disease model.

### Oral NR effectively increases hepatic NAD^+^ levels in rats

**Fig. 3:**
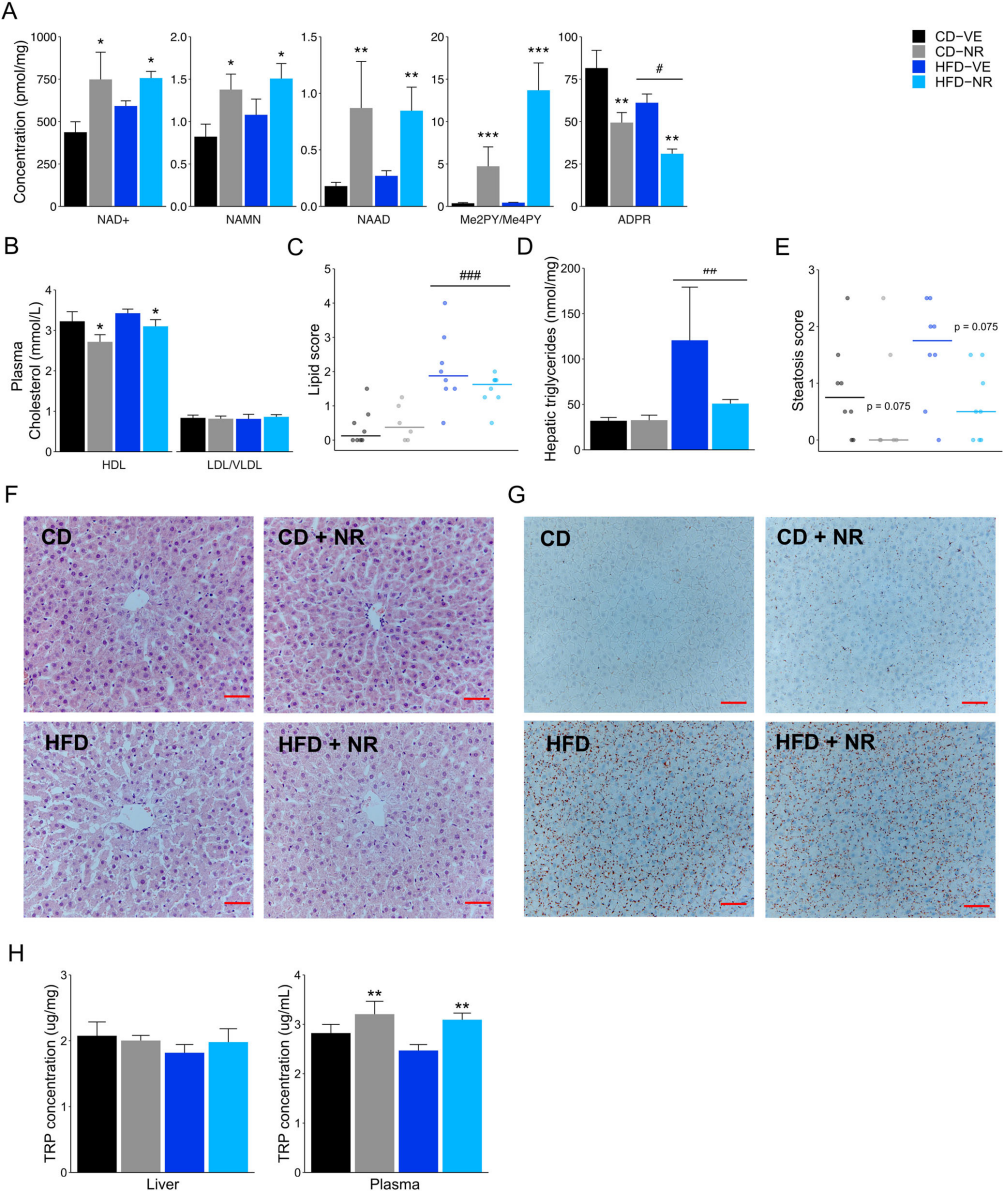
Hepatic NAD^+^-related metabolites, cholesterol measurements, histological grading, triglycerides and tryptophan quantification in rats. **A** Hepatic levels of NAD+, NAMN, NAAD, Me2PY/Me4PY and ADPR. Diet effect: #*p* < 0.05, NR effect: *p < 0.05, **p < 0.01, ***p < 0.001. **B** Plasma levels of HDL and LDL/VLDL. NR effect: *p < 0.05. **C** Lipid score (0–4) shown as median and individual values. Diet effect: ###*p* < 0.001. **D** Hepatic triglycerides quantification. Diet effect: ##*p* < 0.01. **E** Steatosis score (0–3) shown as median and individual values. **F** Representative hematoxylin and eosin (H&E) staining used to assess steatosis score. Scale bar: 100 µm. **G** Representative Oil Red staining used to assess lipid score. Scale bar: 100 µm. **H** Hepatic and Plasma levels of tryptophan (TRP). NR effect: **p < 0.01. Data are shown as mean ± SEM if not otherwise specified. *n* = 6–8. CD control diet, HFD high-fat diet, NR nicotinamide riboside, VE vehicle.

To determine the effects of NR on levels of liver NAD^+^ and related metabolites, we assessed the NAD^+^ metabolome in this tissue. Irrespective of diet, NR treatment significantly increased levels of NAD^+^ as well as NAMN, NAAD, and Me2PY/Me4PY. On the other hand, ADPR decreased with NR in both groups and was generally lower in the HFD group compared to the CD group (Fig. 3A). All other assessed metabolites that did not change with diet or treatment are presented in Table 1.

**Table 1:** Liver NAD^+^ metabolome profile in rats.

	Group mean (pmol/mg)				Main effect of diet			Main effect of treatment		
	CD-VE	CD-NR	HFD-VE	HFD-NR	% change from CD	95% CI	FDR adj. *p*-value	% change from VE	95% CI	FDR adj. *p*-value
**ADPR**	81.6	49.4	61.1	31	**−29.6**	**(−68.9 to −7.3)**	**0.041**	**−43.5**	**(−83.1 to −19.9)**	**0.001**
**Me2PY/Me4PY**	0.4	4.7	0.4	13.7	104.7	(8.1 to 287.6)	0.11	**1447.1**	**(718.7 to 2823.6)**	**3.39E-07**
MeNam	0	0	0	0	16.9	(−17.2 to 65)	0.464	−18.3	(−42.1 to 15.2)	0.381
NA	0	0.1	0	0.2	78.2	(−36.6 to 400.6)	0.392	90.5	(−32 to 433.3)	0.381
**NAAD**	0.2	0.9	0.3	0.8	39.2	(−19.5 to 140.7)	0.381	**207.8**	**(78.4 to 431.2)**	**0.003**
NAADP	0	0	0	0	−10.8	(−60.2 to 11.9)	0.517	−18.4	(−68.8 to 5.6)	0.31
**NAD**+	437.4	748.5	591.8	756.6	25.3	(−4.5 to 64.3)	0.259	**45.8**	**(11.3 to 91.1)**	**0.044**
NADP+	117.9	111.7	120.9	134.2	10.9	(−17.2 to 25.7)	0.452	3.7	(−26 to 19.7)	0.73
NAM	372.5	420.8	326.6	333.1	−16.8	(−56.1 to 4.2)	0.286	7.3	(−27.2 to 24.2)	0.573
**NAMN**	0.8	1.4	1.1	1.5	17.8	(−26.9 to 34.9)	0.392	**51.1**	**(27.7 to 58.6)**	**0.044**
NAR	0.8	1.1	0.7	0.9	−10.9	(−89.1 to 16.5)	0.573	36.5	(−19 to 50.3)	0.259
NMN	21.7	30.8	28.4	37	16.7	(−22.2 to 75.1)	0.517	43.8	(−4 to 115.4)	0.246
NR	8.4	11.7	7.7	10.2	−10.7	(−88.8 to 16.6)	0.573	34.8	(−22.2 to 49.2)	0.266

NAD^+^-related metabolites were determined using target LC-MS in liver samples of rats. *n* = 6–8. Significant effects are highlighted in bold.

*CD* control diet, *HFD* high-fat diet, *NR* nicotinamide riboside, *VE* vehicle.

Untargeted mass spectrometry-based metabolomics was performed on plasma to determine the effects of NR supplementation on NAD- and non-NAD-related metabolites in the circulation. As expected, significant relative increases in NAD-related metabolites were seen in NR-supplemented animals and most of these metabolites were not affected by diet (Table 2). N-methyl-2/4-pyridone-5-carboxamide was only detected in samples from one of the diet groups. Further analysis of these data showed an increase in two bile acids, tauro-deoxycholic acid (TDCA) and glycol-cholic acid (GCA), in response to NR treatment (Supplementary Table 1).

**Table 2:** Plasma NAD^+^-associated metabolites in rats.

*m/z*	Metabolite	Diet group	Fold change	*p*-value
139.050	Nicotinamide N-oxide	CD	82	1.58E−05
		HFD	88	3.06E−13
123.055	Nicotinamide (Nam)	CD	25	0.000554
		HFD	35	1.78E−11
137.071	N(1)- methyl nicotinamide (MNA)	CD	13	0.000291
		HFD	11	8.92E−06
138.055	N-methylnicotinate (trigonelline)	CD	4.7	6.92E−05
		HFD	2.9	3.15E−05
256.082	Nicotinate Riboside (NaR)	CD	4.3	0.003053
		HFD	6.2	5.47E−05
153.066	N-methyl-2/4-pyridone-5-carboxamide	CD	–	NS
		HFD	8.2	0.001225

Significant NR effects on NAD^**+**^-associated metabolites in plasma tested within the diet groups. *n* = 6–8.

*NS* non-significant.

As NR has been reported to affect plasma cholesterol composition in mice^48^, we measured HDL and LDL/VLDL cholesterol in plasma. NR reduced HDL cholesterol by 12.3% while LDL/VLDL was unaffected (Fig. 3B). As an increase in bile acid production can affect levels of HDL cholesterol^49^ and may explain the increase in TDCA and GCA levels, the lumen content of bile acid in the cecum was quantified by targeted LC-MS-based metabolomics. The number of samples per group in which the specific bile acid was quantified is shown in Supplementary Fig. 3a. Furthermore, the sum of all identified primary, secondary, unconjugated, and conjugated bile acids are summarized in Supplementary Fig. 3b, while the average abundance of quantified bile acids is shown in Supplementary Fig. 3c. However, no significant changes in the bile acid composition were found.

Monoacylglycerols (MAGs), diacylglycerols (DAGs), ceramides, acylcarnitines or short-chain fatty acids (SCFA) can also affect cholesterol levels^50–55^ and were, therefore, quantified in the plasma by untargeted MS metabolomics (Supplementary Fig. 4a–b). Dihydroxy, tetrahydroxy, and pentahydroxy bile acids, as well as 1-monoacylglycerols and acylcarnitines, increased with HFD feeding. Moreover, butyrate increased significantly with NR, whereas for acetate this was only borderline significant.

NR can prevent diet-induced liver steatosis in mouse models^48^ but this is not a consistent finding^56^. To determine the degree of liver steatosis, lipid accumulation and potential inflammation in response to HFD feeding, we performed histological grading of hematoxylin and eosin (H&E) and Oil Red-O-stainings, and quantified total triglyceride levels in the liver. In addition, we quantified levels of CD45 as an indicator of pro-inflammatory cell infiltration. While we observed an increase in lipid score (Fig. 3C, F) and triglyceride levels (Fig. 3D) in HFD-fed rats, HFD did not induce steatosis in our model (Fig. 3E, G). However, there was a general tendency towards a decrease in steatosis score in the two NR-treated groups (Fig. 3E). Moreover, levels of CD45 did not change significantly with either diet or treatment (Supplementary Fig. 5a–b). Taken together, oral NR supplementation increased hepatic levels of NAD^+^, the two bile acids TDCA and GCA in plasma and decreased HDL levels. NR supplementation also increased levels of the SCFAs acetate and butyrate. However, NR treatment only tended to reduce steatosis with no changes in lipid score, inflammation markers, triglyceride levels, or cecal bile acid composition.

### Oral NR affects plasma but not liver tryptophan levels in rats

Tryptophan (TRP) can be used to generate NAD^+^ through the kynurenine pathway^57^ and levels of TRP in plasma or the liver may change in response to oral NR supplementation. While no changes in TRP were observed in the liver, we found NR increased plasma TRP levels in both diet groups (Fig. 3H) indicating that increased availability of NR or NR-derived NAD^+^ precursors may limit de novo synthesis of NAD^+^ from TRP. Moreover, the observed increased TRP levels were significantly and positively correlated with levels of 3-indoxyl sulfate (*β* = 0.38, *p* = 0.04) and indole propionate (*β* = 0.44, *p* = 0.01), two TRP-derived metabolites previously associated with microbiota metabolism and described to be increased in centenarians^58,59^

### Effects of NR on the activity and abundance of NAD^+^-dependent proteins in rats

To determine whether the observed increased NAD^+^ concentration in the liver had any effects on the abundance or activity of NAD^+^-producing and utilizing enzymes, specific targets were analyzed by Western blot analyses. NR supplementation did not alter the abundances of NAD^+^-producing [nicotinamide riboside kinase 1 (NRK1), NAMPT] or -utilizing enzymes (SIRT1, SIRT3, SIRT5), (Supplementary Fig. 6a), but levels of PARP1 decreased in response to HFD feeding (Supplementary Fig. 6b). Nonetheless, total acetylation levels of lysine motifs of higher molecular weights were significantly increased in response to NR in CD- and HFD-fed animals (Supplementary Fig. 6c–d). To determine whether the increased acetylation levels were due to sirtuin activity, we analyzed acetylation levels of p53 and MDH2, two well-known targets of SIRT1 and SIRT3, respectively. Moreover, we also analyzed total lysine malonylation, as SIRT5 can remove succinyl and malonyl from lysine residues^60^. The protein abundance of p53, but not the levels of acetylated p53 (p53 Ac-K^379^), was affected by both diet and NR treatment (Supplementary Fig. 6e). Consequently, the ratio between acetylated and non-acetylated p53 (p53 Ac-K^379^/p53) was reduced by both diet and treatment (Supplementary Fig. 6e). These data suggest reduced SIRT1 activity. In contrast to p53, MDH2 acetylation was increased in response to both HFD and NR, as well as its total protein abundance. When normalizing acetylated MDH2 to total MDH2 (MDH2 Ac-K^239^/MDH2), there was no effect of diet or NR treatment (Supplementary Fig. 6f). These data also indicate unchanged or even reduced SIRT3 activity. HFD feeding in mice has been demonstrated to decrease lysine malonylation, and NR increased lysine malonylation^56^. In line with these observations, HFD significantly decreased lysine malonylation in rat liver compared to CD-fed animals but we observed no effects of NR (Supplementary Fig. 6g).

Besides SIRT3, carnitine acetyltransferase (CRAT) can act as a modulator of acetylation and regulates the disposal of excess acetyl-CoA^61,62^. Therefore, we measured CRAT levels in the liver. The data displayed a tendency toward an increase of CRAT in the NR groups but did not reach statistical significance (Supplementary Fig. 6h). In plasma, levels of acylcarnitines only increased significantly in HFD-fed animals with no effect of NR (Supplementary Fig. 4b).

Collectively, while NR increased NAD^+^ levels in the liver, NR treatment promoted global acetylation of lysine motifs in both CD- and HFD-fed animals, which suggests increased acetyltransferase or lower deacetylase activities. These results may be related to sirtuin activity, which at least for SIRT1 appeared reduced in response to NR.

### NR supplementation increases energy absorption in HFD-fed rats

**Fig. 4:**
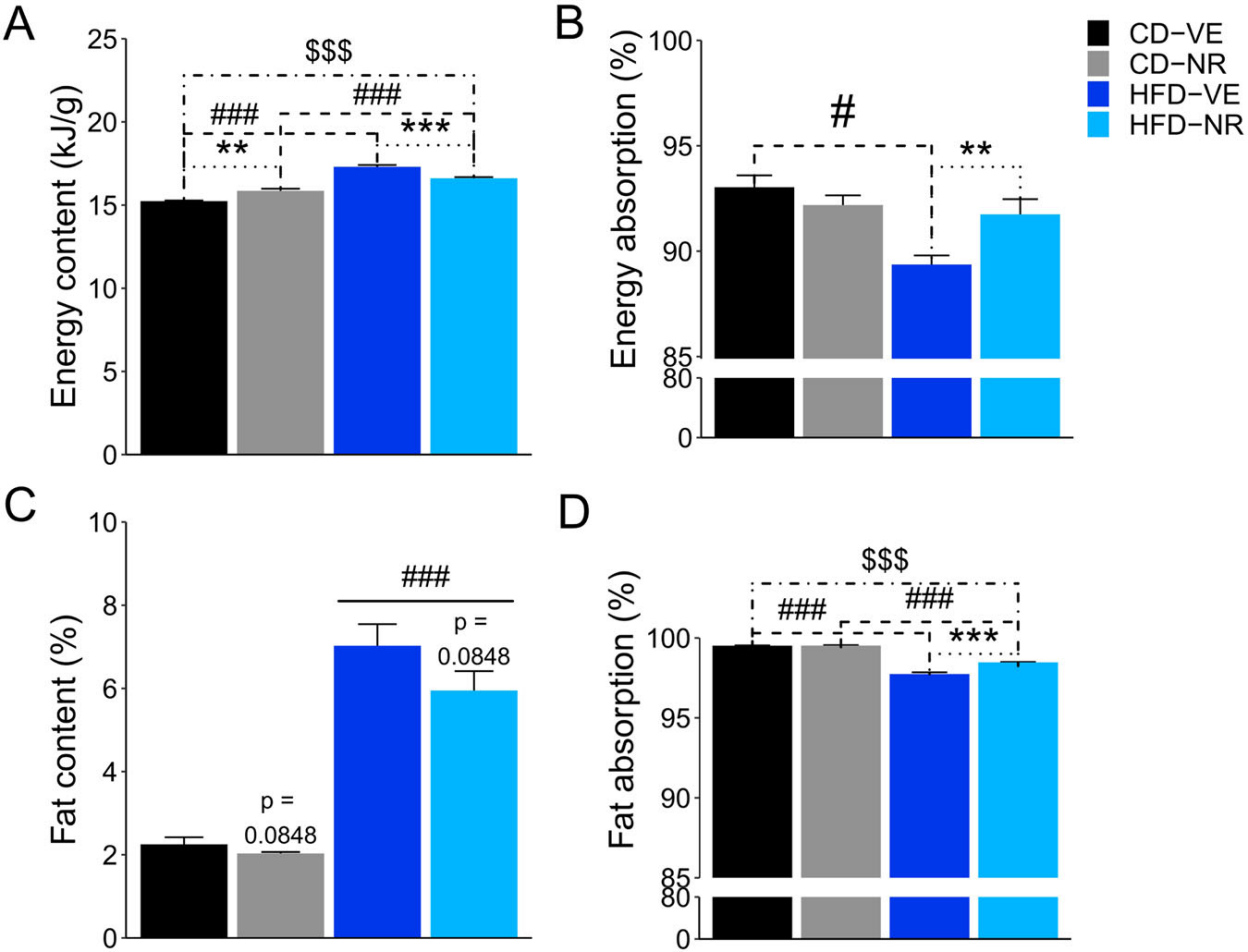
Energy and fatty acid absorption. **A** Energy content of feces and **B** energy absorption. Energy absorption is depicted as the percentage of available energy from feed. Multiple comparison tests: Diet effect: (- - -) #*p* < 0.05, ###*p* < 0.001. NR effect: (^**……**^) **p < 0.01, ***p < 0.001, Interaction effect: (-^.^-^.^-^.^-^.^) $$$*p* < 0.001. **C** Fat content depicted as the percentage of available fat from feed. Multiple comparison tests: Diet effect: ###*p* < 0.001. **D** Fat absorption depicted as the percentage of available fat from feed. Multiple comparison tests: Diet effect: (- - -) ###*p* < 0.001. NR effect: (^**……**^) ***p < 0.001, Interaction effect: (-^.^-^.^-^.^-^.^) $$$*p* < 0.001. Data are shown as mean ± SEM. *n* = 3–4.

To determine whether the changes in the plasma bile acid pool were associated with changes in intestinal lipid uptake, the absorption of energy and fat from food was quantified. The energy content of the feces was increased by HFD (Fig. 4A). Within the vehicle (VE)-treated groups, feces from HFD-fed animals had 13.5% more energy compared to CD-fed. The increase was significantly smaller in NR-treated animals, for which HFD only increased energy content by 4.8% (Fig. 4A). Interestingly, in CD-fed animals, NR increased energy content by 4.1%, whereas in HFD-fed animals, NR reduced the energy content in the feces by 4.0%. Furthermore, we found a 0.9% lower energy absorption in the CD-fed animals treated with NR compared to the CD-fed vehicle group, but a 2.6% increase in energy absorption in the HFD-fed animals treated with NR compared to the HFD-fed vehicle group (Fig. 4B). In addition, we measured the fat content of the fecal samples to determine whether changes in energy content were caused by altered fat absorption. Fecal fat content was 3-fold greater in samples from HFD-fed animals compared to CD-fed animals (Fig. 4C). Moreover, fat absorption was also found to be different between treatment groups (Fig. 4D) with a significant interaction between diet and treatment. HFD-fed animals absorbed a smaller percentage of the available fat than CD-fed animals in both treatment groups. In VE-treated animals, this reduction was 1.8%, and in NR-treated animals, it was 1.0% compared with CD-fed animals. Moreover, NR treatment increased fat absorption in HFD-fed animals by 0.7% but did not affect CD-fed animals. Collectively, these data indicate that in the HFD condition, NR increases energy absorption from the gut. Most, if not all, of this enhanced energy absorption was due to increased fat absorption.

### NR modulates the rat fecal microbiota by inducing a sustained increase in *Erysipelotrichaceae* and *Ruminococcaceae* species

**Fig. 5:**
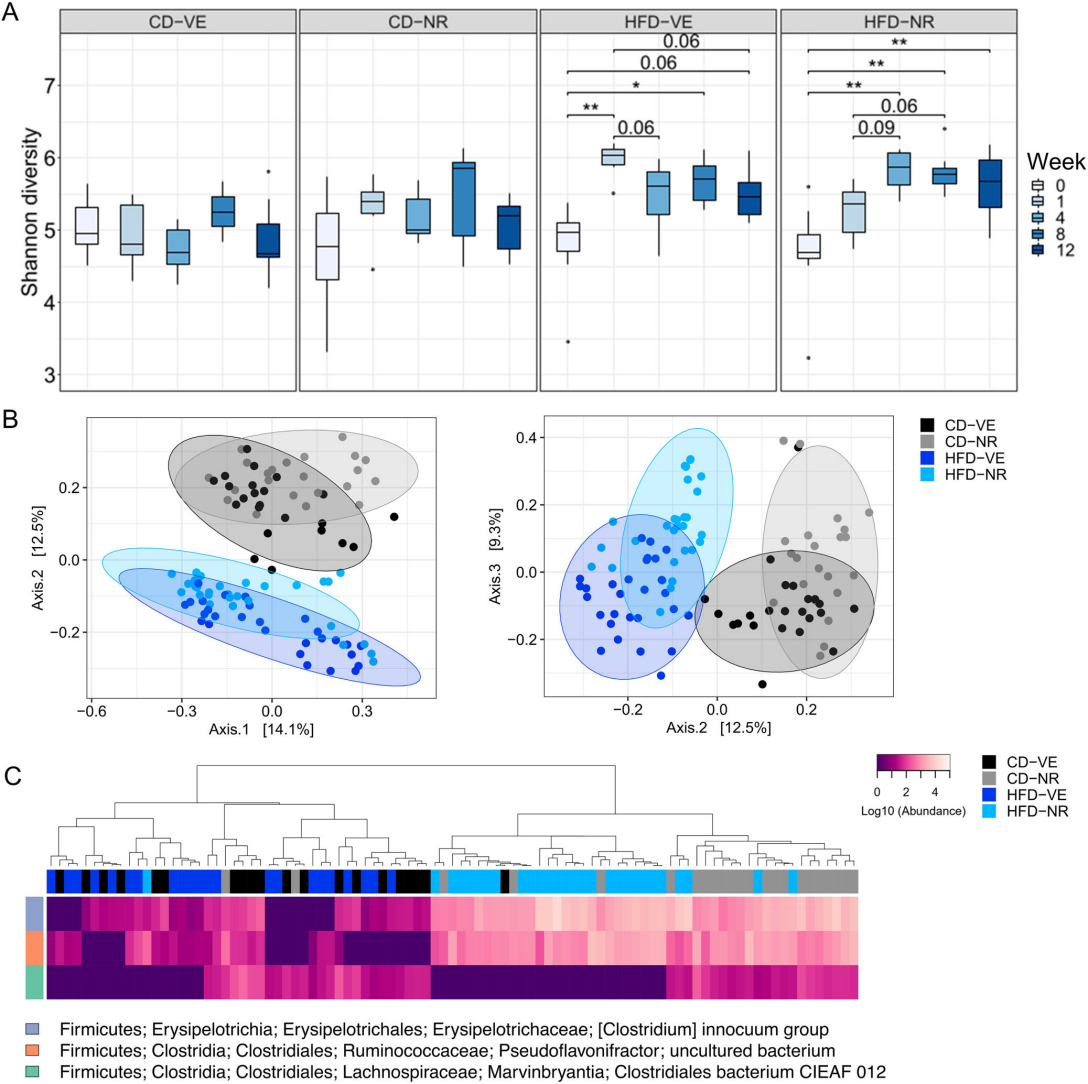
Effects of NR on the rat microbiota diversity. **A** α-diversity calculated by the Shannon diversity index. *p < 0.05, **p < 0.01. Shown as median and quartiles (1^st^ and 3^rd^) and the minimum and maximum by the whiskers. **B** PCoA plots of β-diversity based on Bray-Curtis distance matrix for the study period. Colors are according to diet and treatment groups. Axes indicate the proportion of variance explained. **C** Abundance heatmaps of differentially abundant features at the species level. *n* = 6–8.

Changes in macronutrient absorption are associated with changes in the gut microbiome^63^. Thus, we assessed the effects of NR oral supplementation on the rat gut microbiota composition in fecal samples collected before, three times during, and at the end of the study (Fig. 1). The within-sample diversities were calculated using the Shannon index as an α-diversity metric. There was no significant difference in the control diet groups across all time points, as indicated by the Shannon index (Fig. 5A). Surprisingly, HFD without NR treatment increased α-diversity in weeks 1 and 8 compared to week 0, which contrasts with most^64–66^ but not all^35^ reports of HFD effects on α-diversity. HFD-fed animals treated with NR presented increased α-diversity in weeks 4, 8, and 12 compared to the baseline. Additionally, we observed a borderline significant difference comparing week 1 to weeks 4 and 8.

To further explore microbial community compositions, we identified the top 30 most abundant species on average in all cohorts (Supplementary Fig. 7). Abundance heatmaps showed that the differences between the groups were subtle and there was no major shift in the overall microbial community structure. Next, we performed principal coordinate analysis (PCoA) using Bray-Curtis (Fig. 5B), weighted and unweighted UniFrac, and Jaccard distance matrices (Supplementary Fig. 8a) to investigate between-group diversities (β-diversity), which is a measure of overall changes in microbial community composition. We found that the change in diet from chow to purified diets (week 0 to week 1) significantly affected the overall β-diversity of the bacterial community (Supplementary Fig. 8b, four left panels). Consequently, we excluded the week 0 time point from the rest of the analysis. Examining the time points within the study period, we observed an apparent clustering of samples according to diet and NR-treated groups in most of the tested distance matrices except for CD-VE vs. HFD-NR and HFD-VE using unweighted UniFrac and CD-VE vs. HFD-NR using Jaccard distance matrices according to the PERMANOVA and PERMDISP results summarized in Supplementary Table 2 (Supplementary Fig. 8b, four right panels).

To identify features with significant changes in their abundances, the feature table was collapsed at the species level using a pre-trained SILVA-based Naive Bayes classifier and a differential abundance test was conducted with ANCOM. Differentially abundant species were all within the *Firmicutes* phylum and their abundances are shown by the heatmaps comparing weeks 1–12 in Fig. 5C. We found a sustained increase in two species belonging to the *Erysipelotrichaceae* and *Ruminococcaceae* bacterial families in response to NR treatment. In contrast, another species belonging to the *Lachnospiraceae* family was increased only in CD-fed animals, independent of the NR treatment. Indeed, using a linear mixed effect (LME) model for regression analyses involving longitudinal data (weeks 0–12), we confirmed that the abundances of the *Erysipelotrichaceae* and *Ruminococcaceae* species were significantly impacted by treatment while *Lachnospiraceae* species were significantly impacted by diet (Supplementary Fig. 8C). Adding an interaction term to the model did not change it significantly for any of the species.

### NR affects microbiota composition in the lower intestinal tract of rats

**Fig. 6:**
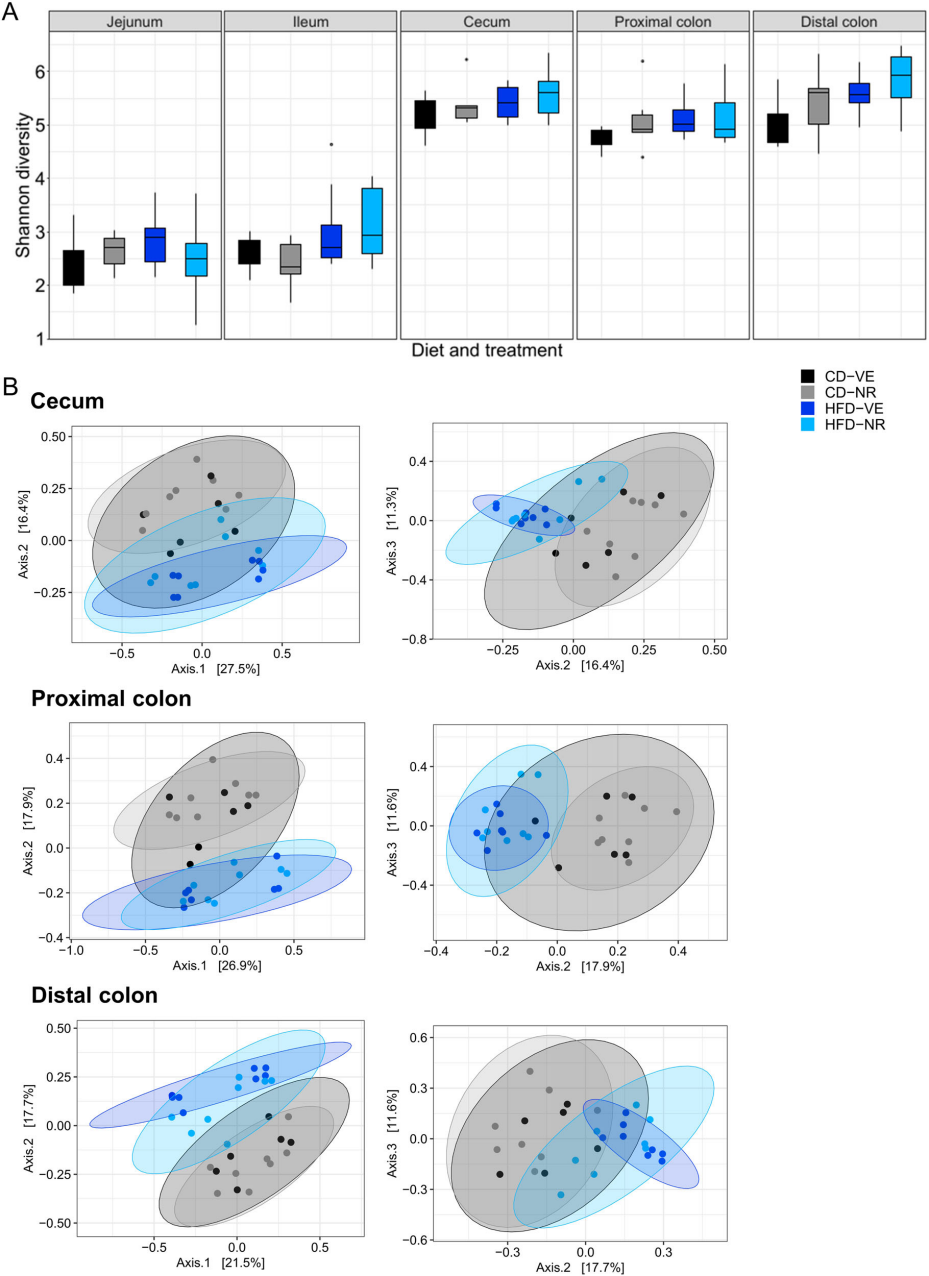
Effects of NR on different intestinal sections of the rat microbiota diversity. **A** α-diversity of individual intestine sections calculated by the Shannon diversity index. Shown as median and quartiles (1^st^ and 3^rd^) and the minimum and maximum by the whiskers. **B** PCoA plots of β-diversity of individual intestine sections based on Bray-Curtis distance for the study period. Colors are according to diet and treatment groups. Axes indicate the proportion of variance explained. *n* = 6–8.

**Fig. 7:**
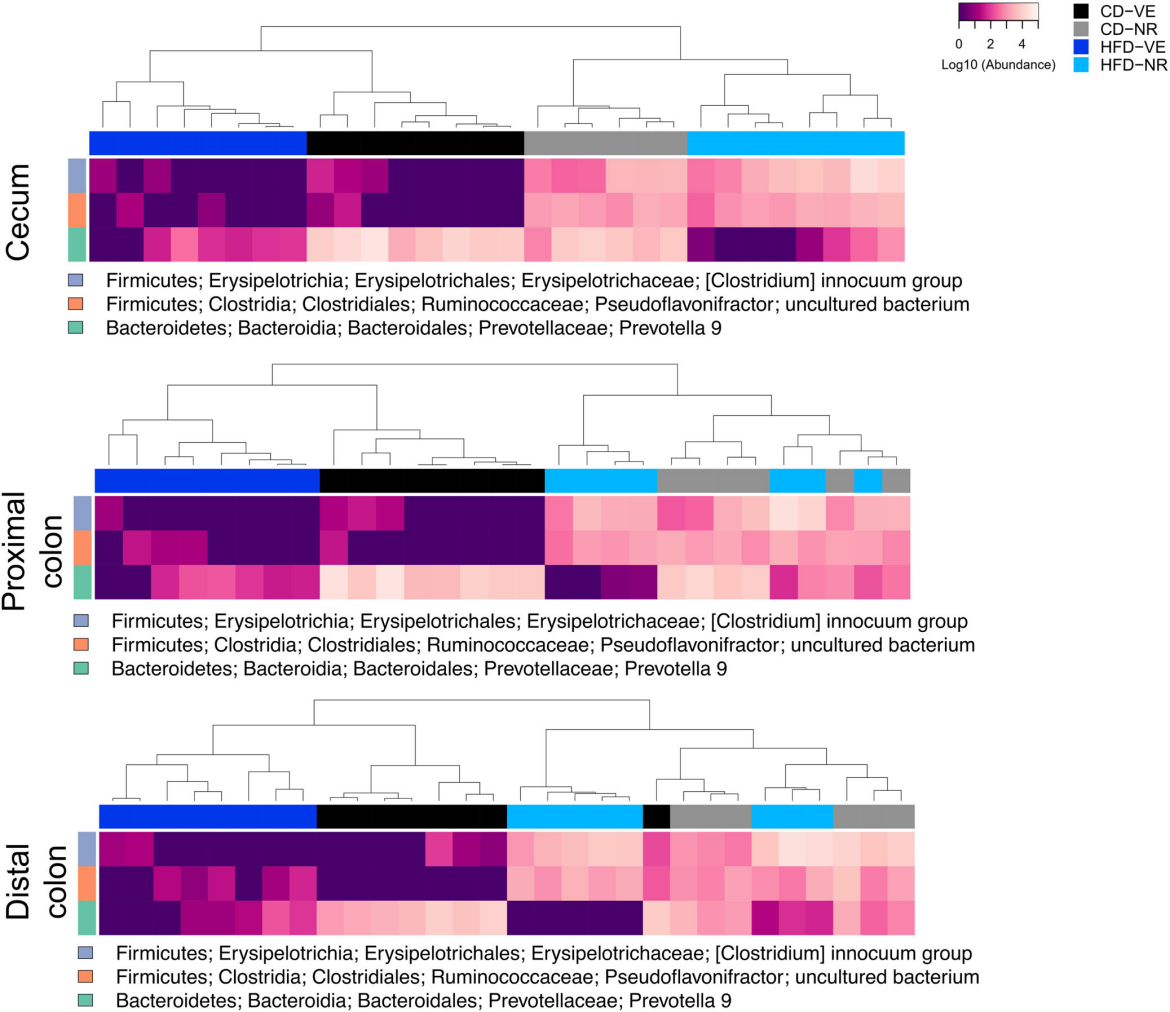
Hierarchical clustering of differentially abundant species within the rat lower intestinal microbiota. Heatmaps of individual intestinal sections based on the abundance of the features identified to be differentially abundant. *n* = 6–8.

The microbiota compositions of the jejunum, ileum, cecum, proximal colon, and distal colon were analyzed separately to investigate the effects of NR in specific parts of the intestinal tract in rats. In this case, the sampling was performed after the euthanization of distinct animals. Repeating the analysis described above, the α-diversity indicated by the Shannon index did not show any significant difference between the cohorts regarding diet, NR treatment, or diet and NR treatment combined in the examined intestinal sections (Fig. 6A). However, the β-diversity based on the Bray-Curtis distance showed significant clustering of samples based on diet in all sections, except for jejunum in the upper intestinal tract and distal colon. The latter showed significant clustering based on NR treatment (Fig. 6B). Clustering patterns in the ileum and proximal colon regarding diet, as well as clustering patterns in distal colon and cecum regarding diet and treatment (except for CD-VE vs. CD-NR in both, and additionally HFD-VE vs. CD-NR and HFD-NR in the latter), presented significant differences as summarized in Supplementary Table 2. Abundance heatmaps of differentially abundant bacteria identified in the lower intestinal tract, namely cecum, proximal colon, and distal colon samples, are presented in Fig. 7. No features were found to be differentially abundant in the upper intestinal parts. In response to NR, all three lower sections showed a significantly increased relative abundance of two species belonging to *Ruminococcaceae* and *Erysipelotrichaceae* families within *Firmicutes* and one species belonging to the *Prevotellaceae* family within *Bacteroidetes*. Taken together, these data show that NR acts to remodel the microbiota composition in the lower intestinal portion.

### NR promotes in vitro growth of PnuC-positive bacteria within *Erysipelotrichaceae* and *Ruminococcaceae*

**Fig. 8:**
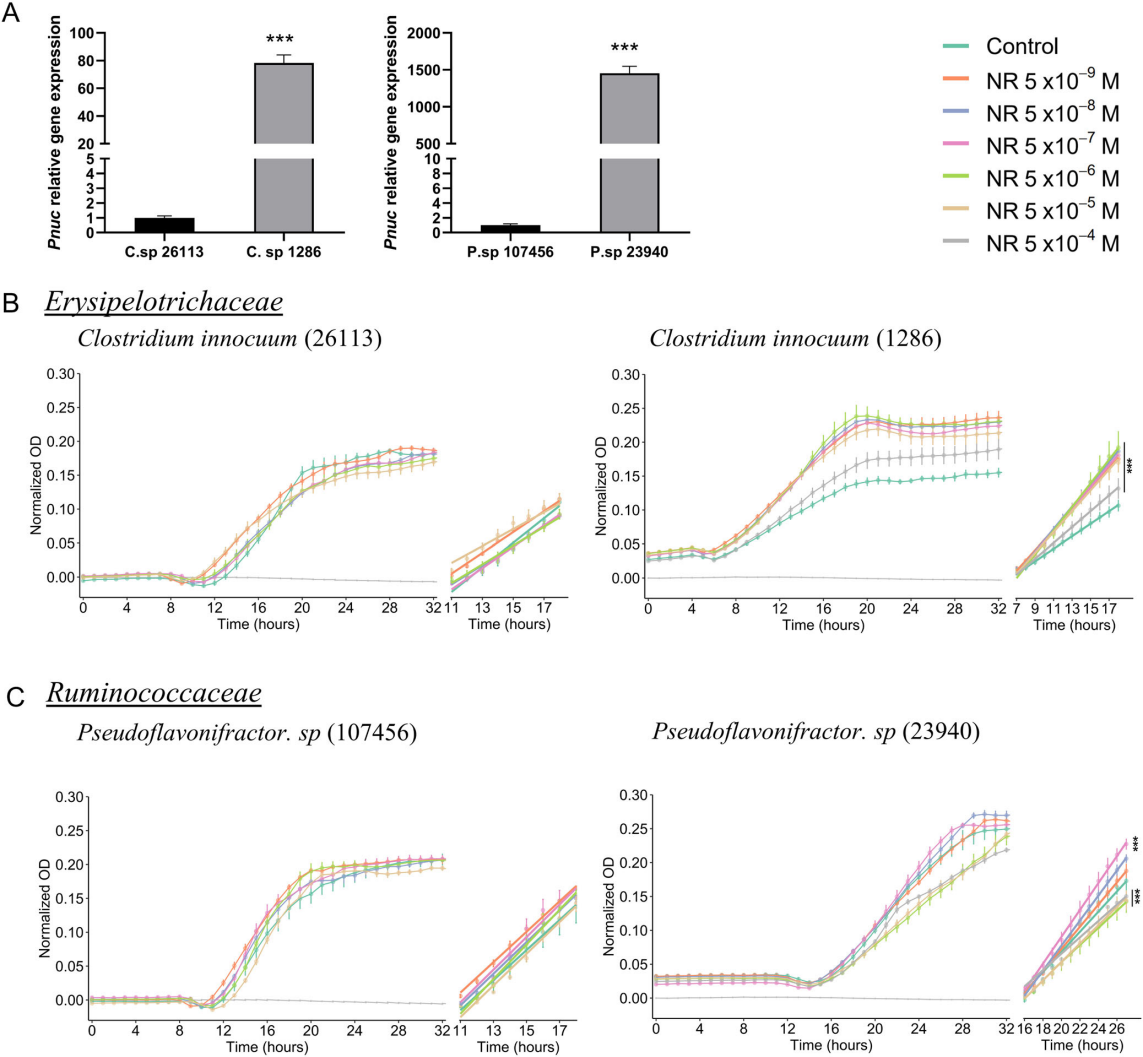
Bacterial *PnuC* gene levels and growth curve. **A** Relative levels of the *PnuC* gene in *Clostridium innocuum* (1286 and 26,113 strain types) and *Pseudoflavonifractor sp* (23,940 and 107,456 strain types) within *Erysipelotrichaceae* and *Ruminococcaceae* families, respectively. Multiple comparison test. ***p < 0.01. Data are shown as mean ± SEM. *n* = 4. **B** Bacterial growth curve and slope calculation of the exponential phase for *Clostridium innocuum* (1286 and 26,113 strain types). ***p < 0.01. **C** Bacterial growth curve and slope calculation of the exponential phase for *Pseudoflavonifractor sp* (23,940 and 107,456 strain types). ***p < 0.01. Data are shown as mean ± SEM of 3 independent experiments. *n* = 8.

Bacteria within *Erysipelotrichaceae* and *Ruminococcaceae* families are known to express the NR transporter *PnuC*^38,67^. Therefore, we hypothesized that NR would promote growth of *PnuC*-expressing bacteria strains within these families compared to control strains without the transporter. In order to address this question, we ran a BLAST search for the enriched Amplicon Sequence Variants (ASVs) identified in our metagenomics sequence data. Out of the BLAST matches, *Clostridium innocuum* (DSM strains 1286 and 26,113 within *Erysipelotrichaceae*) and *Pseudoflavonifractor* sp. (DSM strains 23,940 and 107,456 within *Ruminococcaceae*) strains were commercially available at the German Collection of Microorganisms and Cell Cultures (DSMZ). We confirmed the presence of *PnuC* gene in *Clostridium innocuum* − 1286 and *Pseudoflavonifractor* sp. − 23,940 (Fig. 8A). The other two strains were negative for *PnuC* and therefore used as negative controls. Interestingly, treatment with NR at different concentrations increased bacterial growth rates in the strains which were positive for *Pnuc* when compared to their respective control groups (without NR treatment) (Fig. 8B, C). Moreover, NR had no growth effect on *PnuC*-negative strains of *Clostridium innocuum* − 26,113 or *Pseudoflavonifractor* sp. − 107,456. This latter strain even presented a lower growth rate with NR compared to its control. These data suggest that the increase in abundance of specific bacterial species with NR may be related to the presence of *PnuC* in those species.

### NR modulates the mouse gut microbiota by increasing species within *Lachnospiraceae* and decreasing *Burkholderiaceae* and *Bacteroidaceae* abundances

**Fig. 9:**
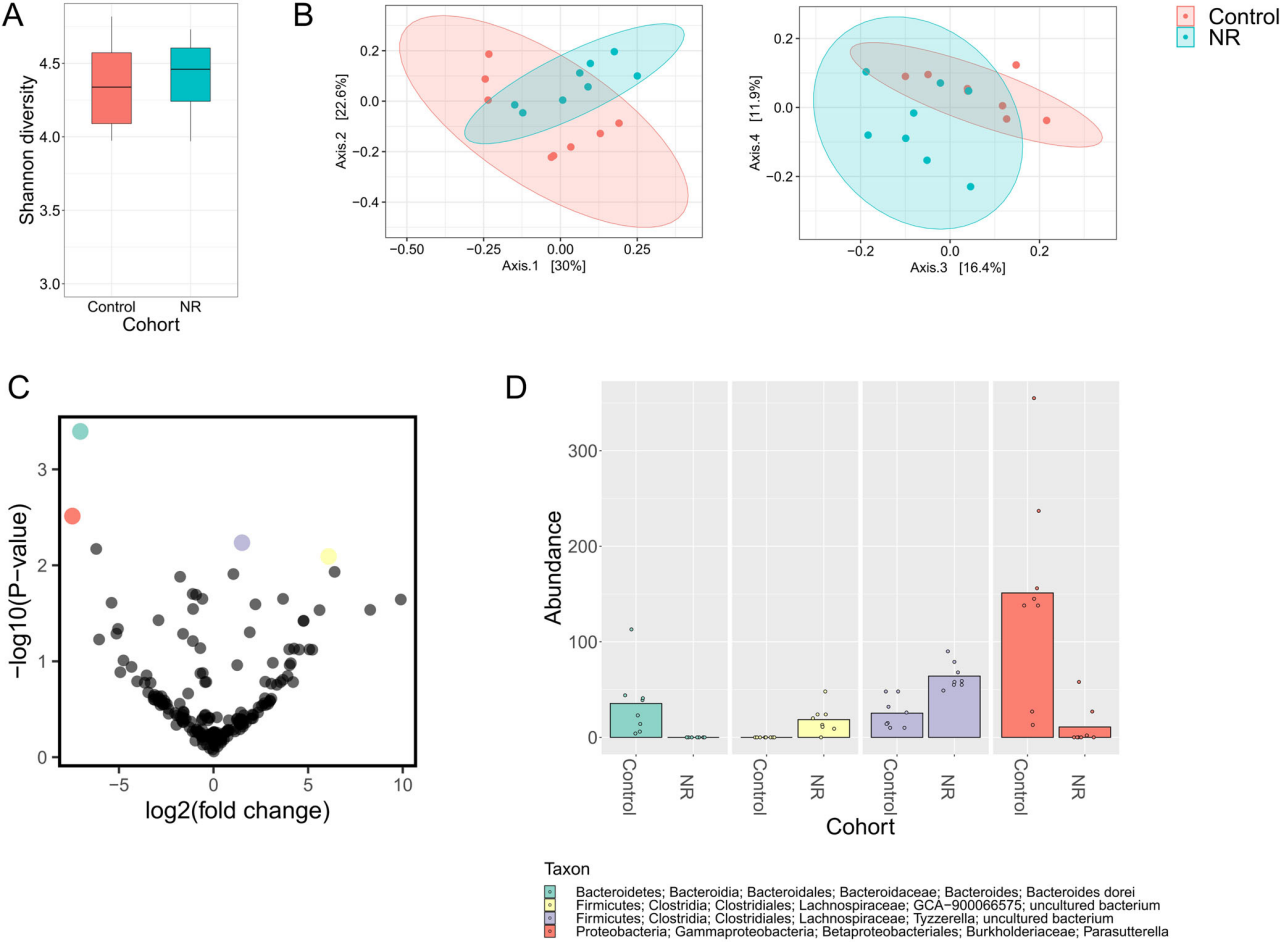
Effects of NR on the mouse microbiota. **A** α-diversity calculated by the Shannon index. Shown as median and quartiles (1^st^ and 3^rd^) and the minimum and maximum by the whiskers. **B** PCoA plot of β-diversity based on Bray-Curtis distance matrix. Colors are according to treatment groups. Axes indicate the percentage of variation explained. PERMANOVA, *p* = 0.006 for clustering based on cohort. **C** Volcano plot displaying *p*-values for abundance fold-change according to ALDEx2 differential abundance test. **D** Abundance of differentially abundant features based on treatment. Samples are grouped according to treatment. *n* = 8.

Since some studies in mice have reported a beneficial effect of NAD^+^ precursor supplementation^20,68^, we investigated whether 12 weeks of 400 mg/kg/day NR supplementation impacts the mouse gut microbiota. There were no significant changes in α-diversity (Fig. 9A). However, the samples were clustered by treatment as shown in the β-diversity PCoA plot based on Bray-Curtis distance (Fig. 9B). Differential abundance test using ANCOM did not reveal any significant changes, therefore we investigated the changing features with ALDEx2, which revealed that while two uncultured species belonging to *Lachnospiraceae* (within *Firmicutes*) were significantly increased in the NR supplemented cohort, *Parasutterella* (within *Proteobacteria*) and *Bacteroides dorei* (within *Bacteroidetes*) were significantly reduced (Fig. 9C, D and Supplementary Fig. 9). Collectively, these data show that NR can also alter the mouse microbiota.

### NR supplementation does not change the human gut microbiota

**Fig. 10:**
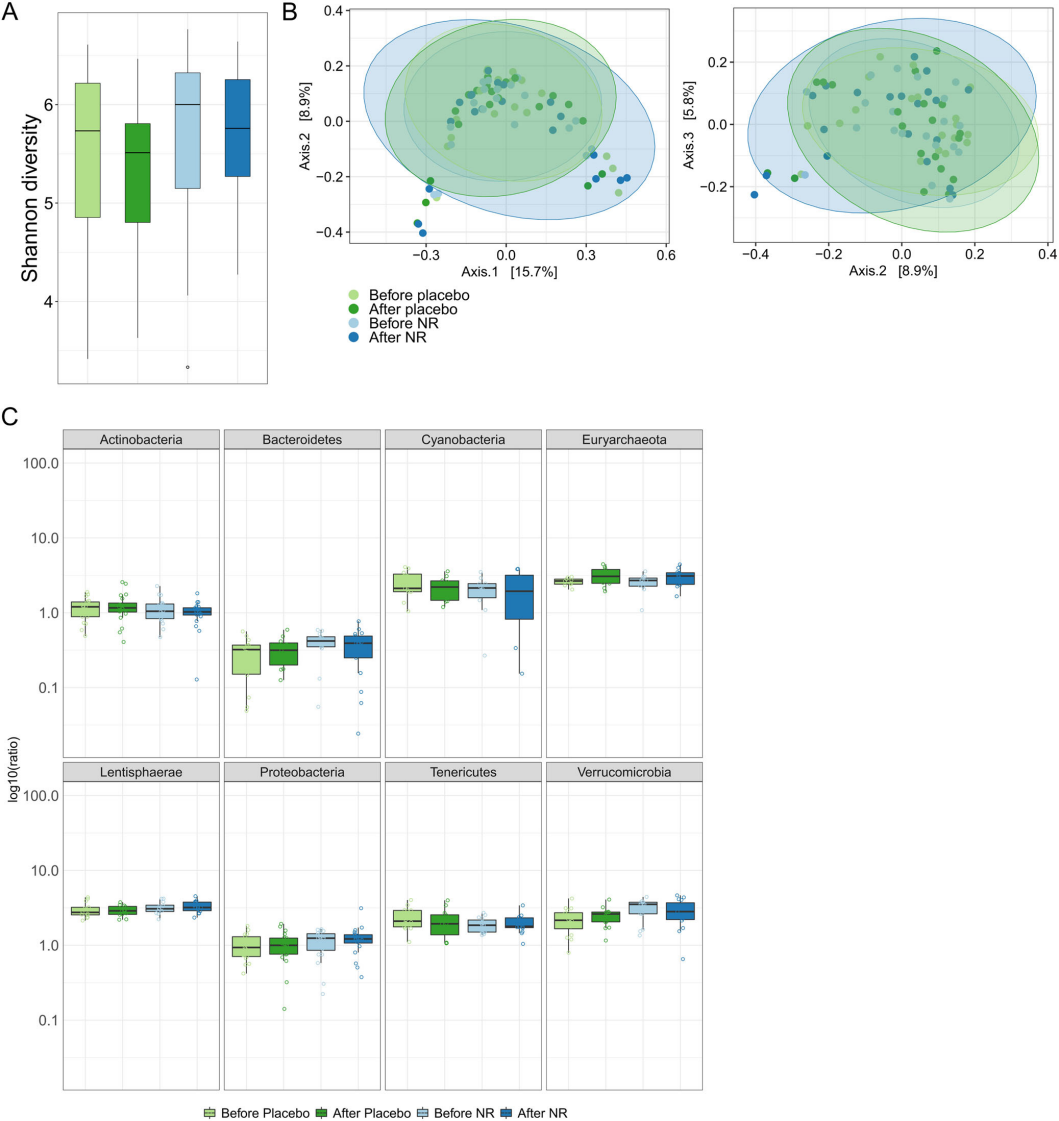
Effects of NR on the human microbiota. **A** α-diversity calculated by the Shannon index. Shown as median and quartiles (1^st^ and 3^rd^) and the minimum and maximum by the whiskers. **B** β-diversity calculated by Bray-Curtis diversity matrix. **C** *Firmicutes* to other phyla ratios after treatment intervention in humans. Shown as median and quartiles (1^st^ and 3^rd^) and the minimum and maximum by the whiskers. *n* = 20 per group.

Forty middle-aged, non-diabetic and obese male participants received either a placebo or NR supplementation (1 g twice daily) over 12 weeks. Stool samples were collected and analyzed before and after the intervention^10^. The 16S rRNA gene profiling analysis showed no significant changes in the Shannon index (Fig. 10A) or β-diversity calculated by Bray-Curtis before and after NR supplementation (Fig. 10B). We noticed that samples from the same participant appeared to be closer together in both placebo and NR cohorts, as shown by the PCoA plots in Supplementary Fig. 10. We did not detect any differentially abundant features at any taxonomic levels. When comparing the ratio of *Firmicutes* (the major abundant phylum) to other phyla, there was no significant difference detected (Fig. 10C). Therefore, NR treatment did not affect human microbiota composition in this setting.

## Discussion

We performed 12-week studies with oral NR supplementation in HFD-induced obese Sprague-Dawley rats, C57BL/6J mice, and middle-aged, obese, insulin-resistant men. In rats, we showed that NR mitigated the HFD-induced obesity phenotype by significantly reducing fat mass with a tendency to decrease body weight. No significant changes with NR were observed regarding metabolic outcomes, such as glucose and insulin tolerance, whole-body energy expenditure, or liver glycogen levels. However, our results indicate that NR tends to reduce liver steatosis scores but at the same time increases energy and fat absorption in the HFD setting. Finally, NR changes the gut microbiota of rats and mice to favor bacterial species capable of synthesizing NAD^+^ from specific precursors, while no such change was observed in humans.

Sprague-Dawley (SD) rats were chosen as a model of diet-induced obesity due to their capacity to develop hyperglycemia, dyslipidemia^69^, and altered microbiome upon HFD^64–66^, but only a few studies have determined the effects of NR treatment in rats^70–72^. We reported that HFD-fed rats gained more weight and fat mass compared to CD-fed animals and were glucose and insulin intolerant, indicating that HFD feeding indeed induced obesity and insulin resistance. HFD-fed rats had a 33% greater total fat mass at the end of the study, which was evenly distributed between visceral and subcutaneous adipose deposits. NR lowered HDL levels in both diet groups while LDL levels were unaffected. This is in line with other observations of NR-mediated reductions of total cholesterol in HFD-fed animals^73^. Remarkably, NR increased energy and fat absorption but only in the context of HFD. This observation needs to be confirmed in future NR studies in both animals and humans to better understand and interpret specific metabolic adaptations.

The liver is a central organ in the whole-body regulation of glucose, lipid, and cholesterol metabolism^74^. The liver NAD^+^ metabolome analysis showed that NR, independently of diet, increased levels of NAD^+^ and its related metabolites NAMN, NAAD, and Me2PY/Me4PY, while ADPR decreased with NR. This response in NAD^+^, NAMN, and NAAD levels to orally delivered NR is consistent with previously reported data from mice^44^, and the increased level of Me2PY/Me4PY indicates increased breakdown of NAM. Previous studies in HFD- and high fat/high sucrose diet (HFHS)-fed mice have shown negative correlations between NAD^+^ and hepatic triglyceride levels^48,73^. However, our data on lipid score, steatosis score, and triglyceride levels did not correlate with the abundance of NAD^+^.

Despite no changes in liver tryptophan (TRP) levels, plasma TRP levels significantly increased in rats treated with NR independently of the diet. Moreover, such an increase was correlated with two TRP-breakdown metabolites, 3-indoxyl sulfate and indole propionate. These metabolites were previously found to be increased in samples of centenarians^58^. Therefore, we speculate that higher availability of NAD^+^ precursors by NR supplementation could result in lower engagement of the kynurenine pathway to produce NAD^+^ while TRP becomes more bioavailable to be converted into 3-indoxyl sulfate and indole propionate, a possible mechanism associated with healthy aging^58,59,75^.

Increased NAD^+^ levels can activate sirtuins without changing their abundance. SIRT1, SIRT3, and SIRT5 levels were unaffected by NR in this study, so we assessed global acetylation status and acetylation of specific sirtuin targets. We found that both the HFD and NR increased global acetylation. Moreover, acetylation levels of the SIRT1 target p53 were unaltered, while acetylation levels of the SIRT3 target MDH2 increased with diet and NR. Increased global acetylation in the liver has previously been demonstrated with oral NAM supplementation^76^ and may be related to the inhibitory effects of NAM on sirtuins. We did not find any changes in NAM levels in the liver, but levels of Me2PY/Me4PY did increase with NR supplementation indicating increased rates of breakdown of NAM. Finally, HFD decreased total lysine malonylation, as well as PARP1 levels, while NR treatment had no effect. Collectively, our data indicate that changes in acetylation status with NR are likely not related to NAM, and the disparity between whole tissue NAD^+^ levels and downstream sirtuin activities requires further investigation.

Before reaching the liver, ingested NR enters the intestinal milieu inhabited by the gut microbiota. Bacteria use NAD^+^ for various metabolic processes. They produce NAD^+^ through de novo synthesis or by utilizing NAD^+^ precursors from the surrounding environment in the amidated and deamidated pathways^38,44^. Bacteria within the *Firmicutes* and *Actinobacteria* phyla depend on NAD^+^ precursors for growth^38^. Moreover, bacterial metabolization of NR could alter the pool of NAD^+^ metabolites available for the host and for the production of other bacterially derived metabolites, such as secondary bile acids and SCFAs. In fact, it was recently shown that gut bacteria convert NR to NA via deamination of NAM^44^. Based on this, we hypothesized that NR may serve as a selection factor for these bacterial phyla. In response to NR treatment, the rat fecal 16S rRNA gene profiling data showed an overall alteration in the bacterial community. Specifically, two species belonging to the *Erysipelotrichaceae* and *Ruminococcaceae* families within the *Firmicutes* phylum increased with NR. An NR-specific transporter, *PnuC*, has previously been described in *Firmicutes*^38,67^. Moreover, a BLAST search against the UniProt database search engine (www.uniprot.org/blast/) found the *PnuC* gene to be present in members of both *Erysipelotrichacea* and *Ruminococcaceae*, indicating that these bacteria likely use NR. The BLAST search for the enriched ASVs identified in our metagenomics sequence data followed by qPCR confirmed the presence of the NR transporter *PnuC* gene in *Clostridium innocuum* (strain 1286) and *Pseudoflavonifractor* sp. (strain 23940). Furthermore, treatment with NR at different concentrations increased the growth rates of these strains. Conversely, strains that are negative for *PnuC* and were used as negative controls, did not respond to NR treatment. Finally, an analysis of the luminal content of different intestinal sections showed that the abundance of two species belonging to *Erysipelotrichaceae* and *Ruminococcaceae* were increased in response to NR in the lower intestinal portions, including the cecum and the proximal and distal colon. These findings could indicate a spatial preference for NR utilization.

The unconjugated form of TDCA, deoxycholic acid (DCA), is produced from cholic acid (CA) in an NADH- or NADPH-dependent reaction by bacteria-expressed homoserine dehydrogenase (HSDH) enzyme found in all major phyla, such as *Firmicutes* and *Actinobacteria*^27,77,78^. We found an increased concentration of the tauro-conjugated, secondary bile acid TDCA in plasma in response to NR treatment. As such, increased concentrations of TDCA may be a result of modulation of the bacterial community and a higher abundance of phyla capable of producing TDCA. DCA has been described as a Farnesoid X receptor (FXR) agonist^79^, suggesting that alterations in the microbial community and the production of secondary bile acids can increase FXR activity by DCA stimulation and consequently reduce plasma cholesterol. Such a connection has previously been reported in germ-free rats, where the absence of bacteria leads to a reduction of the bile acid pool in plasma, liver, and other tissues^80^. In our study, however, we do not find any correlations between levels of plasma TDCA and GCA and plasma HDL or between plasma TDCA and GCA and plasma LDL/VLDL. This suggests a potential lack of causality between the changes in the bile acids and cholesterol. Therefore, further investigation is necessary to confirm whether NR treatment affects cholesterol levels in rats.

*Erysipelotrichaceae* abundance is associated with diet-induced obesity^36,81–83^ and altered energy uptake from the intestines^84^. *Erysipelotrichaceae* abundance increased in response to a low-fat diet (LFD) and a HFD supplemented with the non-digestible fiber hydroxypropyl-methylcellulose, but not in response to HFD alone. Moreover, these diets showed genus-level differences within the *Erysipelotrichaceae* family^85^. The *Erysipelotrichaceae* family consists of four closely related lineages (M1-M4), the abundances of which have been found to depend on diet and genotype. Healthy, chow-fed wild-type and apolipoprotein knockout (Apoa-I^−/−^) mice primarily harbor the M1 and M3 lineages, while HFD-feeding leads to a shift to M2 and M4 lineages. As such, abundance changes of the different lineages may be diet-related rather than a consequence of host phenotype^82^. An intriguing observation in our study was that in the context of HFD and NR, energy and fat absorption was enhanced. This was associated with the increased *Erysipelotrichaceae* abundance in the same treatment group and is in line with previously published results showing that elevated abundance of *Erysipelotrichaceae* increases energy absorption^84^. It is interesting to note that despite the enhanced energy and fat absorption in the HFD-NR group, compared to the HFD-VE group, fat mass decreased in these animals. As we did not detect changes in whole-body energy expenditure between these two groups and since lean body mass even tended to be reduced in NR-treated animals, we cannot readily account for this disparity in energy balance.

Untargeted metabolomics of SCFA in the plasma of rats showed a significant NR effect in butyrate and a borderline effect in acetate levels. Acetate is the most abundant fecal SCFA^86^ and it is capable of inducing protein acetylation as an epigenetic modification^87^. Furthermore, elevated acetate levels are potentially beneficial for cardiometabolic disease risk^88^. The borderline effect of NR on acetate appears in line with increased acetylation levels in the liver. In addition, acetate levels positively correlate with *Ruminococcaceae* abundance^88^. Therefore, this suggests that the NR-mediated enrichment of *Ruminococcaceae* may increase levels of acetate and subsequent acetylation in target tissues.

The mouse microbiota was affected after 12 weeks of NR treatment, but the observed changes were different from those observed in the rats. Differences in animal species, diet, environmental factors as well as the source of NR could account for these different responses. NR increased *Lachnospiraceae* (*Firmicutes* phylum) species. Species belonging to the *Lachnospiraceae* family (e.g., *Eubacterium halli*) have been reported to be important butyrate producers found in the gut microbiota. Unlike the rats, the acetate and butyrate levels were unaltered in mice (data not shown), which could be due to a slighter effect of NR on the abundance of the regulated species. SCFAs such as butyrate are capable of inhibiting intestinal inflammation, maintaining intestinal barrier integrity, and participating in gut motility regulation^89^. Butyrate production may have protective endocrine functions with regard to insulin sensitivity and glucose tolerance through the production of GLP-1 and GLP-2^90,91^. Moreover, the presence of *Eubacterium halli* in obese and diabetic mice (i.e., *db/db* model) showed an increase in energy metabolism and insulin sensitivity without consequent effects on body weight and food intake or side effects regarding toxicity^92^.

In humans, the metagenomics data did not show a significant effect of NR on the microbiota composition. Although there were no significant changes in the Shannon index (α-diversity) or taxonomic abundance profiles between different groups (β-diversity), the ratio of *Firmicutes/Proteobacteria* in the NR group was significantly increased after the treatment when compared to the placebo with contingency-based filtering of 5%, keeping the features present in at least 5% of the samples. However, since we have set a threshold of 10% in our pipeline, this effect has no longer reached significance. *Firmicutes* are positively related to the production of bile acids and, consequently, an increase in fatty acid absorption^84^. Therefore, a potential increase of *Firmicutes* rather than *Proteobacteria* due to scavenging of resources and environmental competition could be a positive effect of NR administration. However, this is still speculative and needs further investigation.

Patients diagnosed with cirrhosis have shown a decrease in the abundance of non-pathogenic bacteria, such as *Lachnospiraceae*, *Ruminococcaceae*, and *Clostridiales XIV*, which was associated with a reduction in pro-inflammatory endotoxins. On the other hand, pathogenic bacteria, such as *Enterococcus*, *Enterobacteriaceae*, and *Bacteroidaceae* were found to increase^93–95^. The beneficial effects, as well as the different mechanisms involved in the effects promoted by *Lachnospiraceae* were recently summarized^96^.

*Firmicutes*, including *Ruminococcaceae* and *Lachnospiraceae* members, decrease in abundance during aging with consequent replacement by other subdominant genera^97^. NR supplementation was previously shown to rejuvenate aged gut adult stem cells via an NAD^+^/SIRT1/mTORC1 pathway^98^. Therefore, the upregulation of *Firmicutes* in an NR-dependent manner may have potential anti-aging effects to keep a youthful microbiota that can maintain intestine integrity. Furthermore, NR treatment in mice led to decreased *Parasutterella* (*Proteobacteria* phylum) and *Bacteroides dorei* (*Bacteroidetes* phylum). This could be explained by the increase of *Firmicutes* mediated by NR. On the other hand, *Proteobacteria* species have been linked to inflammation and aspects of the metabolic syndrome^99,100^. This is in line with available literature showing the protective effects of NAD^+^ precursor supplementation on metabolic diseases such as obesity and diet-induced liver damage by improving mitochondrial function, glucose tolerance, and serum cholesterol^8,9,36,101^. Furthermore, *Bacteroides dorei* was only present in VE-treated animals, demonstrating that NR could suppress the growth of this bacterial taxa. There are only a few studies relating the role of *Bacteroides dorei* to metabolism. Still, a Finnish study showed that this taxon dominates the gut microbiome before autoimmunity in children at high risk for type 1 diabetes (T1D), suggesting that an increase in *B. dorei* abundance is a potential indicator of T1D development^102^.

It is important to realize that the discrepancy in the results observed across species could be attributed to various factors. For example, rats and mice received a daily dose of 300 mg/kg and 400 mg/kg NR in drinking water, respectively. Moreover, the nutritional status of rodents could be affected due to differences in animal facilities. Humans, on the other hand, received a daily dosage of 2 g of NR (1 g twice daily) or a placebo, which was comprised of microcrystalline cellulose. In addition, external factors influencing humans, such as smoking habits and physical activity, are more difficult to control. Lastly, circadian rhythm could also contribute to the obtained data, since rodents are known for being active in the dark phase, which is different from human behavior.

Collectively, this 12-week study on the metabolic and microbial effects of NR treatment in the HFD-induced obese Sprague-Dawley rats reduced fat mass and tended to reduce lean and total body weight. Moreover, NR induced increased energy and fat absorption exclusively in the HFD context. These changes occurred without changes in whole-body metabolism, despite increased plasma and liver levels of NAD^+^ metabolites. In the rat gut microbiota, we observed a consistent increase in the abundance of *Firmicutes*, including species belonging to *Erysipelotrichaceae* and *Ruminococcaceae* in response to NR treatment. These species are known to promote fat and energy absorption. Thus, the enhanced fat and energy absorption observed in response to NR treatment may be mediated by changes in the gut microbiome. Our data also suggested an inverse correlation between *Firmicutes* and *Proteobacteria* in humans, although this observation was not statistically significant. In mice, species within *Firmicutes* were also increased while *Proteobacteria* and *Bacteroides dorei* were decreased in response to NR. These findings indicate that oral NR supplementation modifies the gut microbiota in certain animal species and may serve as a selection factor for bacteria within NAD^+^ precursor-dependent taxa.

## Methods

### Rat data

#### Housing, diet and supplementation

Male Sprague-Dawley (SD) rats (11–12 weeks of age) were obtained from Envigo (Italy) and acclimatized to the animal facility for 4–5 weeks prior to the experimental period. The rats were kept in individually ventilated cages with a constant temperature of 22 °C at a 12/12 light/dark cycle at the Faculty of Health and Medical Sciences, University of Copenhagen. Thirty-two male SD rats were divided into four groups (*n* = 8) based on body weight upon arrival and were allowed 4 weeks of acclimatization on a fortified chow diet (1319, Altromin) and water before the study started. During the 12-week study period, the animals were fed an experimental 60% HFD (D12492, Research Diets, Inc.) or a matched control diet (CD) with 10% fat (D12450J, Research Diets, Inc.) ad libitum in combination with 300 mg/kg/day nicotinamide riboside (NR, NIAGEN®, ChromaDex) diluted in drinking water, which was used as vehicle (VE). We have previously determined that NR is stable in drinking water^56^. During this period, the animals were subjected to an oral glucose tolerance test (oGTT, week 6) and an insulin tolerance test (ITT, week 7). We collected fecal samples at five time points, as well as intestinal content from distinct gastrointestinal tract sites at termination for 16S rRNA gene sequencing. Therefore, for fecal samples, the animals were sampled repeatedly and for intestinal samples, the measurements were taken from distinct samples. In addition, we monitored body weight gain weekly, body composition every second week, gas exchange, and feed intake in metabolic chambers (week 12). NR supplementation was adjusted to fluid intake (0.9 is an added factor to account for any spill from water bottles, estimated to a 10% loss) and body weight on cage-basis (2 animals/cage) three times a week (Supplementary Fig. 11) using the formula:

$$dosage_{cage}\left( {mg/mL} \right) = \frac{{Weight_{cage}\left( {kg} \right) \cdot \,300\,mg/kg/day}}{{Solution\,intake_{cage}\left( {mL/day} \right) \cdot \,0.9}}$$

The experimental setup is summarized in Fig. 1. One animal had to be euthanized during the oGTT because the gavage was not executed correctly and one animal was excluded from the study at the time of sacrifice after discovering a tumor in the lower torso. Both animals were from the CD-NR group. Tumor development was likely independent of treatment as Sprague-Dawley rats are prone to spontaneously develop tumors^103^. None of the data from these two rats have been included in the analyses. The experiment was approved by the Danish Animal Experimental Inspectorate and conducted following the EU convention for the protection of vertebrate animals in scientific experiments under license no. 2012-15-2934-152.

#### Body weight, body composition, and fat deposits

Body weight was measured weekly in a non-fasting state. Body composition was assessed by MRI scan (EchoMRI™, USA) every other week. Fat deposits [subcutaneous, visceral (gonadal), and brown adipose tissues] from one side of the animal were weighed at the time of sacrifice. Body weight and fat mass were analyzed as the area under curve (AUC) controlled for the baseline measurement.

#### Oral glucose tolerance test (oGTT) and insulin tolerance test (ITT)

Glucose tolerance was tested by oral gavage of a 50% glucose solution to reach a glucose dosage of 2 g/kg body weight in 6-h fasted animals. Blood glucose response was measured on tail blood using Contour XT glucometer (Bayer). Insulin response was measured on plasma from tail blood collected in EDTA-coated tubes and stored on ice until centrifugation at 8400 × *g* for 6 min at 4 C. Insulin concentration was measured using Mercodia Rat Insulin ELISA (Mercodia AB) following the manufacturer’s protocol. Homeostatic Model Assessment of Insulin Resistance (HOMA-IR) was calculated as HOMA-IR = (FPG + FPI)/135, where fasting plasma glucose (FPG) is in mmol/L and fasting plasma insulin (FPI) is in mU/L. The constant of 135 is based on an average value of 1 for healthy young animals^104^. Insulin tolerance was tested by intraperitoneal injection of human insulin at a dosage of 0.75 UI/kg body weight (Actrapid, Novo Nordisk) in 4-h fasted animals. Blood glucose response was measured on tail blood using Contour XT glucometer (Bayer). Human insulin concentration was measured on tail blood (treated as above) using Mercodia Human Insulin ELISA (Mercodia AB) to ensure correct administration of insulin.

#### Energy and fat content/absorption

Fecal samples were collected from the cages over five days (four 24-h periods) in week 8. Daily energy and fat absorption were estimated as the fraction of energy/fat excreted to the energy/fat intake using the formulas:

$$Absorption_{Energy}\left( \% \right) = \left( {1 - \frac{{Energy_{feces}\left( {kJ/g} \right)\,\cdot \,Weight_{feces}\left( g \right)}}{{Energy_{feed}\left( {kJ/g} \right)\,\cdot \,Weight_{feed}\left( g \right)}}} \right) \cdot 100\%$$

Where *Energy*_*feces*_ is the energy content of fecal dry matter, *Weight*_*feces*_ is the amount of feces collected per day in dry weight *Energy*_*feed*_ is the energy content of the feed as given by the manufacturer (Research Diets, Inc.), and *Weight*_*feed*_ is the amount of feed consumed per day in week 12 (sum of the two animals in each cage).

$$Absorption_{Fat}\left( \% \right) = \left( {1 - \frac{{Fat_{feces}\left( {\% fat/g} \right)\,\cdot \,Weight_{feces}\left( g \right)}}{{Fat_{feed}\left( {\% fat/g} \right)\,\cdot \,Weight_{feed}\left( g \right)}}} \right) \cdot 100\%$$

Where *Fat*_*feces*_ is the fat percentage in feces, *Weight*_*feces*_ is the amount of feces collected per day, *Fat*_*feed*_ is the fat content of the feed as given by the manufacturer (Research Diets, Inc.), and *Weight*_*feed*_ is the amount of feed consumed per day in week 12 (sum of the two animals in each cage).

#### Indirect calorimetry

Gas exchange (VO_2_ and VCO_2_) was measured using open-circuit indirect calorimetry (Phenomaster, TSE Systems) over 60 h after a 96-h acclimation period. The animals were single-housed during this period and had monitored *ad libitum* access to feed and water. The respiratory exchange ratio (RER) was calculated as VCO_2_/VO_2_ and energy expenditure was calculated based on the VO_2_ uptake by the Phenomaster software. The packages RAIN (version 1.32.0)^105^, DODR (version 0.99.2)^106^ and CompareRhythms (version 1.0.1)^107^ in R were used to assess differences in rhythmicity. Using RAIN, we determined that all three measurements (i.e., RER, feed intake, and energy expenditure) showed rhythmicity with significant p-values below the threshold of 0.05. We then used DODR to determine whether there was a difference in the rhythms between the two treatment groups for each diet separately. We conducted the DODR analysis at both 1-h and 3-h intervals, as 1-h intervals enabled higher precision. However, as we were only able to conduct the RAIN analysis at 3-h intervals, the interpretations of these results should be considered with care. We found a significant difference in RER rhythmicity in response to NR treatment in HFD-fed animals at both 1-hour (*p* = 5.91 × 10^−7^) and 3-h (*p* = 0.048), while there were no differences in food intake or energy expenditure in the HFD groups and no difference in any of the measurements for the CD groups. Furthermore, to determine how RER differed between the two treatment groups in HFD-fed animals, we used CompareRhythms, a package implementing multiple methods for the analysis of rhythmicity. When using the DODR method from CompareRhythms at 3-h intervals we found no difference between groups while using the limma method at 1-h intervals we found a significant difference in phase by four hours (*p* < 0.001).

#### Tissue, serum, and histological staining

On the sacrifice day, the animals were fasted for 4 h, sedated using 3.5% isoflurane gas, and subjected to cardiac puncture. Blood was extracted using EDTA pre-treated syringes and aliquoted to EDTA-coated tubes for either snap freezing in liquid nitrogen (whole blood) or storage on ice and subsequent centrifugation at 8400 × *g* for 6 min at 4 °C (plasma). Following cardiac puncture and animal sacrifice, hypothalamus, liver, intestinal lumen content, skeletal muscles, and fat deposits were collected in the stated order and snap-frozen in liquid nitrogen. All samples were stored at −80 °C until the day of analysis. Histological processing of the liver samples was performed by the Department of Biomedical Sciences, University of Copenhagen. For H&E staining, tissue samples were fixed and stored in 4% PFA at 4 °C until embedding in paraffin. 4 µm sections were cut on microtome, dewaxed and stained with hematoxylin and eosin (H&E). Qualitative scoring of steatosis was performed on blinded H&E stained slides by two independent histologists using a grading scale of 0–3 points based on the number, size, and distribution of lipid droplets in the hepatocytes. For CD45 immunostaining, fixed samples were incubated overnight with anti-CD45 antibody (1:1600, Abcam) followed by detection with diaminobenzidine (DAB) incubation. CD45 signal was quantified using Zen Blue (Zeiss, Germany) software and calculated as the percentage of positive stained tissue. For Oil Red O (ORO) staining, samples were snap-frozen in liquid nitrogen and stored at −80 °C until cryostat sectioning. The slides were fixed with formalin, stained with ORO and lightly counterstained with hematoxylin. Lipid content was similarly scored on blinded slides by two independent histologists using a grading scale of 0–4 points depending on the distribution, number, size, and intensity of red stainings. 0 was attributed to the lowest grade (no red staining) and 4 to the highest score (widespread, intensive red staining).

#### Hepatic triglyceride quantification

Hepatic triglycerides were extracted by saponification in ethanolic KOH as previously described^108^ with modifications. Briefly, 100–200 mg of tissue was incubated overnight at 55 °C in 350 µL of ethanolic KOH (2:1 ethanol: 30% KOH) followed by adding 650 µL of 50% ethanol. Samples were centrifuged at 13,000 × *g* for 5 min, and 200 µL of the supernatant was recovered to a new tube and containing 215 µL of 1 M MgCl_2_. Triglyceride levels were quantified through colorimetric assay according to the manufacturer’s instructions (KA0847, Abnova, Taiwan). Absorbance signal at 570 nm was determined using a Hidex Sense microplate reader (Hidex, Finland).

#### Glycogen quantification

For hepatic glycogen extraction, 25 mg of crushed liver tissue was heated at 95 °C for 2 h in 500 µL of HCL 1 M for hydrolyzing glycogen to glucose. Samples were then neutralized in 250 µL of NaOH 2 M, centrifuged at 16,000 × *g* for 20 min at 4 °C, and the supernatant was transferred to a new tube. The glucose content was determined by adding to each sample a reaction mix (200 mM Tris·HCl, 500 mM MgCl_2_, 5.2 mM ATP, 2.8 mM NADP) and a 1:1 mixture of 148 µg of a hexokinase and glyceraldehyde 3-phosphate dehydrogenase (pH 7.4). All samples were incubated in a shaking incubator at 22 °C for 15 min and 400 rpm. Glycogen signal was measured by absorbance using a 340 nm filter (Hidex Sense, Finland) and quantified using a glucose concentration curve (1 mg/mL to 0.1 mg/mL) as the standard.

#### Liver and plasma tryptophan levels

Tryptophan levels were quantified using a colorimetric enzyme immunoassay (Novus Biologicals, USA) following the manufacturer´s instructions. Tryptophan was extracted from liver tissue as described below (see western blot section) and values were adjusted according to the total protein concentration. Plasma tryptophan was quantified in undiluted samples.

#### Plasma cholesterol concentrations

Cholesterol fractions were measured on plasma samples using HDL and LDL/VLDL Cholesterol Assay Kit (Abcam plc., USA) following the manufacturer’s protocol.

#### Liquid chromatography-mass spectrometry for NAD^+^ metabolome

Cryogenically frozen liver tissue was crushed using CPO2 impactor and TT05 tissue bags (Covaris, USA). Pulverized sample was transferred to a cryogenic vial and the exact mass was registered. All volumes of extraction solvents were calculated and adjusted to the exact weight of each sample. 380 µL of 4:1 methanol: water (v:v) and 20 µL of an internal standard (IS) solution prepared in methanol were added to the tissue. IS was comprised of the following components with concentration of 10 mg/L.: nicotinic-d_4_ acid, nicotinamide-d_4_, N-methylnicotinamide-d4, D-tryptophan-d5 (CDN isotopes, QC, Canada), β-NAD-d_4_, adenosine-^13^C_5_ (TRC, Toronto, Canada) and adenosine-^13^C_10_,^15^N_5_ 5′-triphosphate (Merck, Darmstadt, Germany).

The cryotube vials for each sample were vortexed and sonicated (45 kHz) on ice for 15 min. The suspension was kept for additional 15 min on ice for protein precipitation. After that, samples were transferred to new 1.5 mL tubes and centrifuged for 3 min at 11,292 *g*, 4 °C. 200 µL of supernatant was collected and dried out using a stream of nitrogen (15 L/min, 45 min, room temperature). Dried extracts were resuspended in 50 µL of methanol: water (4:1; v-v). All samples were stored at −20 °C until further use. An additional 100 µL of the extract was collected and pooled for quality control purposes. A dilution series of standards composed of reported analytes were prepared equally as samples and used for quantitation.

Detection and chromatographic separation were performed on liquid chromatography-tandem quadruple mass spectrometer (UPLC – MS/MS, Waters, USA). Acquity Premier (BEH Amide, 1.7 μm × 2.1, 150 mm, Waters, USA) was used for separation of metabolites. Mobile phases and gradient were selected to enable HILIC separation. Both mobile phases A (water) and B (acetonitrile: water, 9:1; v-v) contained 10 mM ammonium acetate and 5 µM of medronic acid. The mobile phase gradient started with 5% of mobile phase A and it was kept stable for 1 min followed by an increase to 55% during the course of 14 min. Additional 5 min were used to re-equilibrate the gradient to initial conditions. Flow-rate was set to 200 μL/min and column temperature was maintained at 40 °C with an injection volume of 1 μL. Samples were ionized by electrospray ionization (ESI) in positive mode. Metabolite intensities were acquired using multiple reaction monitoring (MRM) mode. Acquisition parameters can be found in Supplementary Table 3. Data were extracted using TargetLynx processing software (Waters, USA). Metabolites were normalized to internal standards and calculated with a calibration curve. Results were expressed in pmol/mg of tissue.

#### Protein and Western blot analyses

Proteins from liver were extracted through tissue homogenization using Tissuelyser II (QIAGEN) and lysis buffer as previously described^109^. Protein concentration was measured using Pierce™ BCA Protein Assay Kit (Thermo Fisher Scientific). Protein homogenates were prepared in Laemmli buffer in 2 µg/uL solutions and denatured by heating. All blots or gels derive from the same experiment and they were processed in parallel. Protein aliquots were loaded in a balanced manner with samples from all experimental conditions present on all gels. 20 µg of protein was run through a hand-casted 5–15% polyacrylamide gel and transferred to ethanol-activated PVDF membranes using a semi-dry system (Amersham Bioscience). Membranes were incubated with the following antibodies: anti-NRK1 (1:1000, Santa Cruz sc-398852), anti-NAMPT (1:2000, Bethyl A700–058-T), anti-SIRT1 (1:1000, Millipore 07–131), anti-SIRT3 (1:1000, Cell Signaling #2627), anti-SIRT5 (1:000, Cell Signaling #8779), anti-pan-malonyl-lysine (1:4000, Cell Signaling #14942), anti-acetyl lysine (1:1000, Cell Signaling #9441), anti-acetyl-Lys^379^-p53 (1:1000, Cell Signaling #2570), anti-p53 (1:1000, Sigma SAB5700817), anti-acetyl-Lys^239^-malate dehydrogenase (MDH2) (custom made, 1:1000, CapraScience, Sweden), anti-MDH2 (custom made, 1:1000, Capra Science, Sweden), anti-PARP1 (1:1000, Cell Signaling #9542), anti-CRAT (1:1000, ThermoFisher # 15170–1-AP) and anti β-actin (1:10.00, Cell Signaling #4970). Protein abundance was detected by luminescence using HRP-conjugated secondary antibodies (Bio-Rad) on a ChemiDoc^TM^ XMR system (Bio-Rad) and quantified using Image Lab software (version 5.0, Bio-Rad, USA). Band intensity of individual samples was normalized to the band intensity of an internal control of mixed liver samples loaded twice on all gels or by the levels of β-actin.

#### Plasma metabolome

Plasma samples were prepared by deproteinization with methanol followed by the addition of methyl tert-butyl ether (MTBE). Water was added after 1 h incubation with shaking (1970 × *g* at 4 °C). Samples were centrifuged and both organic and aqueous layers were transferred to fresh autosampler vials. Pellets were resuspended at 60 °C and supernatants were combined with the aqueous layer. All fractions were dried by speed vacuum. Before analysis, samples were resuspended in either IPA/MeCN/Water (2:1:1) (for the organic phase) or milli-Q water (for the aqueous layer). Equal volumes of samples were pooled for internal control and injected through the queue. Samples were placed in an auto-sampler at 4 °C. Analytes from the organic and aqueous layers were separated using Phenomenex Luna Omega Polar C18 and Amide columns, respectively, and analyzed in both positive and negative ion mode using a Bruker Impact II QTOF. Extracted ion currents were generated using XCMS. Any analyte with an RSD ≥ 0.25 or isotopic peaks was removed. Differentially regulated ions were detected using MetaboAnalyst 5.0 and inspected visually to confirm appropriate peak shape and intensity. Bile acids identified by untargeted metabolomics were confirmed using tandem mass spectrometry (MS/MS).

### FIA-MS for lipid classes and SCFAs

Untargeted identification and quantification of plasma metabolites were conducted using flow-injection mass spectrometry (FIA-MS). Briefly, 50 mL of the plasma samples were used for metabolite extraction. Polar metabolites were extracted using 4:4:2 acetonitrile:methanol:water HPLC grade solution and non-polar metabolites were extracted using MTBE/methanol HPLC grade solution. Collected samples were dried out in speed-vacuum at 37 °C and the pellet was reconstituted in either 100% LCMS pure water or 5:5 LCMS pure water/methanol for polar and non-polar metabolites, respectively. A total volume of 2 mL of the samples was injected by the autosampler twice in the form of two technical replicates. Water was used as blank to subtract background noise. Data processing, normalization, and metabolite identification were carried out using OpenMS^110^ and SmartPeak^111^ frameworks. All downstream statistical analysis was performed using MetaboAnalyst 5.0^112^. In the case of comparison of specific metabolic classes between the cohorts; the normalized intensities of the identified metabolites in each class were summed to represent the whole chemical class. Two-way ANOVA was followed considering diet and treatment as the main effects in R 4.0.2.

#### Targeted analysis of cecum bile acids by LC-MS/MS

Cecum content was prepared by suspending 11–40 mg of sample in either 1 or 2 mL of LCMS grade methanol, followed by vigorous vortexing and shaking at room temperature for 10 min at 1,970 *g*. Samples were then diluted 1:20 in a final volume of 50 μL containing deuterated bile acids. Samples were analyzed and quantified using liquid chromatography-mass spectrometry as described^113^.

#### Sampling of fecal material, gDNA Extraction and 16 S rRNA gene sequencing

Samples were collected at five time points (3 days prior to the study start, after 5 days, and 4, 8, and 12 weeks after the study start). Animals were single-housed during collection and fecal samples were collected hourly over 6 h and stored on dry ice. Intestinal section samples from jejunum, ileum, cecum, proximal colon, and distal colon were collected on the day of sacrifice and snap-frozen in liquid nitrogen. All samples were stored at −80 °C until DNA extraction. Total gDNA was extracted using NucleoSpin® Soil Kit (Macherey-Nagel) according to the manufacturer’s instructions with a few modifications. Briefly, 180–200 mg sample was weighed in tubes containing ceramic beads. Sample lysis was done at RT for 2 × 3 min with 10 min incubation after each round. gDNA from jejunum and ileum was further purified by DNeasy PowerClean Pro Cleanup Kit (QIAGEN) to remove PCR inhibitors. gDNA concentration and quality were measured using Nanodrop2000 (Thermo Scientific) and Qubit (Invitrogen), respectively, before storage at −20 °C. For fecal samples, the V4 hypervariable region of the 16S rRNA gene was targeted using custom-designed primers (TAG Copenhagen, Supplementary Table 4) in a PCR reaction using 5 PRIME HotMasterMix (Quantabio) with 3% DMSO. Amplicon product size and quality were assessed by agarose gel electrophoresis before purification with Agencourt® AMPure® XP (Beckman Coulter). The purified product was diluted to equal concentrations before pooling. Sequencing was performed using the Illumina MiSeq System with MiSeq Reagent Kit v2, 500 cycles (Illumina, Inc.) following the manufacturer’s protocol. Total gDNA from the intestinal sections was shipped to BGI Genomics Co., Ltd (China) for Meta 16S rRNA gene V4 region library preparation and PE250 sequencing with 10 K reads per sample on HiSeq2500 (Illumina, Inc.).

### Mouse samples

#### Housing, diet, and NR supplementation

C57BL/6J male mice were obtained from the UNICAMP Multidisciplinary Center for Biological Research (São Paulo, Brazil). Upon arrival, the mice were acclimatized for four weeks prior to the experimental period. Animals were kept under a constant temperature of 22 °C at a 12/12 light/dark cycle with standard chow and drinking water ad libitum. At 3 months of age, the diet was changed for AIN93M^114^ and all mice were divided into experimental groups. NR was provided by Elysium Health (New York, USA) and added to the drinking water for 12 weeks to provide a daily dose of 400 mg/kg^56^. Water bottles were changed every third day. All mouse measurements were taken from distinct samples. Mouse experiments were approved by the Animal Ethics Committee of the Biology Institute, University of Campinas (UNICAMP), Brazil, under the number 4791–1/2018. Animal maintenance and handling were done according to the International Guiding Principles for Biomedical Research Involving Animals (Council for Laboratory Animal Science, Geneva, Switzerland).

#### Sampling of fecal material, gDNA extraction, and 16S rRNA gene sequencing

Fecal samples from the cecum were collected on the sacrifice day and snap-frozen in liquid nitrogen and stored at −80 °C until DNA extraction. Total gDNA was extracted as described above and shipped to BGI Genomics Co., Ltd (China) for Meta 16S rRNA gene V4 region library preparation and PE250 sequencing with 10 K reads per sample on HiSeq2500 (Illumina, Inc.).

### Human samples

#### Study design, diet, and supplementation

All human data were obtained from a previous randomized placebo-controlled clinical trial that determined the effects of NR supplementation on insulin sensitivity, substrate metabolism, and body composition^10^. In brief, obese (BMI 33.3 ± 0.6 kg/m^2^), sedentary (<30 min exercise per day), and middle-aged (age 60 ± 2.0) males were randomized into NR (*n* = 20) or placebo (*n* = 20) groups and received 12 weeks of supplementation with 1 g of NR twice daily (NIAGEN®, ChromaDex, USA) or placebo (microcrystalline cellulose). Samples were derived from the same individual twice. Furthermore, the study participants were instructed not to change dietary habits during the trial. Dietary intake was obtained as a dietary record, which is a prospective open-ended assessment method, where participants record all foods and beverages consumed over a specific period. All participants in this study received extensive instructions both written and oral on how to fill out the dietary record. Data were obtained prior to the intervention from three days food records and analyzed by trained personnel. The dietary data for the single subject were presented as a mean intake of the three registered days. The data from the food records were analyzed with Vitakost Pro, which is an online database linking to the Danish Food Composition Database - Frida version 4. All data were separated into two treatment groups to evaluate potential differences. Total energy intake was expressed in kilo joule per day (KJ/d; Supplementary Fig. 12a). Energy distribution deriving from macronutrient sources was presented as percentage of total energy intake (TEI; Supplementary Fig. 12b). The distribution of macronutrients was expressed in gram (g; Supplementary Fig. 12c) and daily fat intake was both expressed as percentage of the distribution of fatty acids (%; Supplementary Fig. 12d) and in gram (g; Supplementary Fig. 12e). Participants were not on prescription drugs and were not allowed to take any additional dietary supplements during the study. The study was conducted in accordance with the Declaration of Helsinki after approval by the local Research Ethics Committee (H-3–2014–130) and the Danish Data Protection Agency (1–16–02–714–14). The study was registered at clinicaltrials.gov (NCT02303483) before recruitment was commenced. Participants received oral and written information before written consent was obtained.

#### Sampling of fecal material, gDNA extraction, and 16S rRNA gene sequencing

Fecal samples from participants (80 samples in total—each person sampled repeatedly) were obtained 1–2 days before the beginning of the placebo/NR supplementation and 0–3 days prior to the final dose of placebo/NR, which was given in the morning of the last visit to the laboratory. Samples were collected and immediately frozen at −20 °C and stored at −80 °C until DNA extraction. The dietary patterns of the individual study participants were similar during the fecal sampling periods before and after the intervention based on a 3-day diet journal obtained by each participant. Total gDNA was extracted as described above and shipped to BGI Genomics Co., Ltd (China) for Meta 16S rRNA gene V4 region library preparation and PE250 sequencing with 10 K reads per sample on HiSeq2500 (Illumina, Inc.).

### Metagenomics analysis

Raw DNA sequences were imported into QIIME2 (version qiime2-2020.2) and quality filtering was performed using the DADA2 plugin to detect and correct for sequencing errors, filtering out phiX reads, chimeric sequences, and trimming the sequences based on Phred score plots^115^. A feature table was created for each cohort and diversity analysis was performed. Since some diversity metrics require phylogenetic relationships between the features, we constructed a rooted phylogenetic tree using MAFFT alignment^116^ and the fasttree2 pipeline^117^. α-diversity was calculated using the Shannon diversity index^118^ and compared using the two-sided Wilcoxon signed-rank test with Benjamini-Hochberg p-value adjustment. Additionally, for β-diversity comparisons, Bray-Curtis, Jaccard, weighted and unweighted UniFrac distance matrices were used^119,120^. Normalization of data was done through rarefaction to adjust for variation in sequencing depth and contingency filtering was used to filter out features that are present in less than 10 percent of the samples. The results of the β-diversity calculations were visualized on a PCoA plot for each distance matrix and clustering patterns of the data were investigated and tested using PERMANOVA and PERMDISP^121^. All PERMANOVA and PERMDISP results are provided in Supplementary Table 3. Differentially abundant features between the NR-treated and placebo/vehicle groups were identified using ANCOM for rats^122^ and ALDEx2 comparing two groups in the case of mice and human data^123^ in QIIME2 and R. Linear mixed effect models were also used to model the abundance of the differentially abundant features in rats for longitudinal data to assess the effect of diet and treatment as fixed effects, and week and cage as random effects. In all cases adding an interaction term between the fixed effects did not change the model significantly based on ANOVA results comparing the two mentioned models.

### Bacterial samples

#### PnuC prediction and bacteria culture

The presence of the *PnuC* gene in species within *Erysipelotrichaceae* and *Ruminococcaceae* was verified by running a BLAST for the gene in the NCBI database showing that some members of the enriched families did have the transporter. *Clostridium innocuum* (strains DSM 26113 and DSM 1286) and *Pseudoflavonifractor sp* (strains DSM 107456 and DSM 23940) were purchased from the German Collection of Microorganisms and Cell Cultures GmbH (Braunschweig, Germany). All bacterial strains were cultured in Brain heart infusion media (Oxoid, Basingstoke, UK) and under anaerobic conditions (85% N_2_, 10% CO_2_ and 5% H_2_ at 37 °C in an anaerobic chamber (Coy Laboratory Products, Michigan, USA).

#### Quantitative reverse-transcriptase PCR

*PnuC* gene levels were analyzed by qPCR. Total RNA was extracted using RNeasy Kit (Qiagen, USA) from whole bacteria according to the manufacturer’s instructions. Total extracted RNA was quantified in NanoDrop 2000 spectrophotometer (Thermo Fisher Scientific). cDNA was produced using High-Capacity cDNA Reverse Transcription Kit (Thermo Fisher Scientific). 1 µg of cDNA [TG1] was used for qPCR reaction using Brilliant II Ultra-Fast SYBR Green QPCR Master Mix (Agilent Technologies, Santa Clara, CA, USA). Levels of mRNAs were normalized to 16S gene levels using the ΔΔCT method. Primers for the PnuC promoter were: forward TCATGGATCGAAGCGGTAGG; reverse CAAGGTGACGTTGATCAGGC. Primers for the 16S promoter were: forward ACTCCTACGGGAGGCAGCAG; reverse ATTACCGCGGCTGCTGG.

#### Bacterial growth rate assay

To assess bacterial growth rate, the strains *Clostridium innocuum* (strains DSM 26113 and DSM 1286) and *Pseudoflavonifractor sp* (strains DSM 107456 and DSM 23940) were inoculated at an optical density (OD 595 nm) of 0.001 in 96 well flat-bottom microplates (Corning, Inc, USA). The plates were covered with their lid and incubated at 37 °C in a plate reader (Epoch2, BioTek, USA) placed inside the anaerobic chamber. As treatment, NR was added to some of the wells in concentrations ranging from 10 uM to 1 nM. Bacteria that did not receive any treatment were used as a control and the wells containing sterile media were used as the blank. The OD measurement at 595 nm was carried out every 30 min over a period of 48 h.

### Statistical analysis

All analyses were performed in the R Studio software (R version 4.0.2). Linear regression and one sample *T*-test were used for continuous data. Due to the non-linear nature of body weight and fat mass changes over the study period (weeks 1–12) and variability between animals, we calculated the area under the curve (AUC) and corrected it for baseline as a measure of weight and fat mass gain. Log2-transformation was used when appropriate to ensure equal variance and normal distribution of residuals. Wilcoxon’s exact test was used for the analysis of discrete data. Interactions were analyzed using Tukey’s Honest Significant Difference (HSD) method for multiple comparison tests. All data are reported as Mean ± Standard Error of Mean (SEM) unless otherwise specified. All statistical tests were conducted as two-sided tests with a significance threshold of 0.05. Full descriptions and details of the tests performed are available upon request.

## Supplementary information

Supplementary Figures and Tables

## Data Availability

The datasets analyzed during the current study are available from the corresponding author upon reasonable request. In addition, all 16S rRNA gene sequencing results from rats, mice, and humans have been uploaded to SRA and can be reached via the project number PRJNA945364.
