# Supplementary material for: Oral supplementation of nicotinamide riboside alters intestinal microbial composition in rats and mice, but not humans
Source: NPJ Aging. 2023 Apr 3;9(1):7. doi: 10.1038/s41514-023-00106-4 (PMC10070358; doi:10.1038/s41514-023-00106-4)
Supplement: Supplementary file 1 — Supplementary Figures and Tables [file 41514_2023_106_MOESM1_ESM.pdf]

Supplementary Figure 1

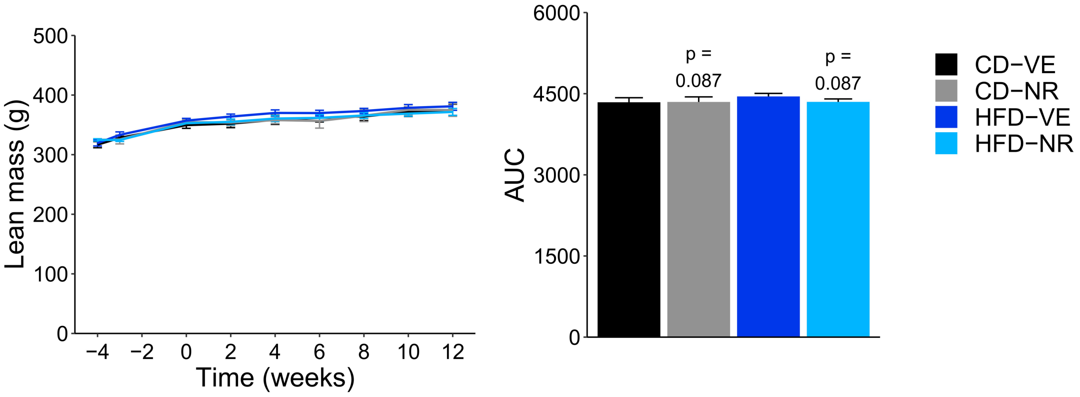

Supplementary Figure 1: Lean mass over the study period
Lean mass curve over the 12 weeks study period (study start) with 3-4 weeks acclimatization period and the average lean mass gain over 12 weeks. Differences in lean mass were analyzed for each week separately and shown as mean ± SEM. n = 6-8.

Supplementary Figure 2

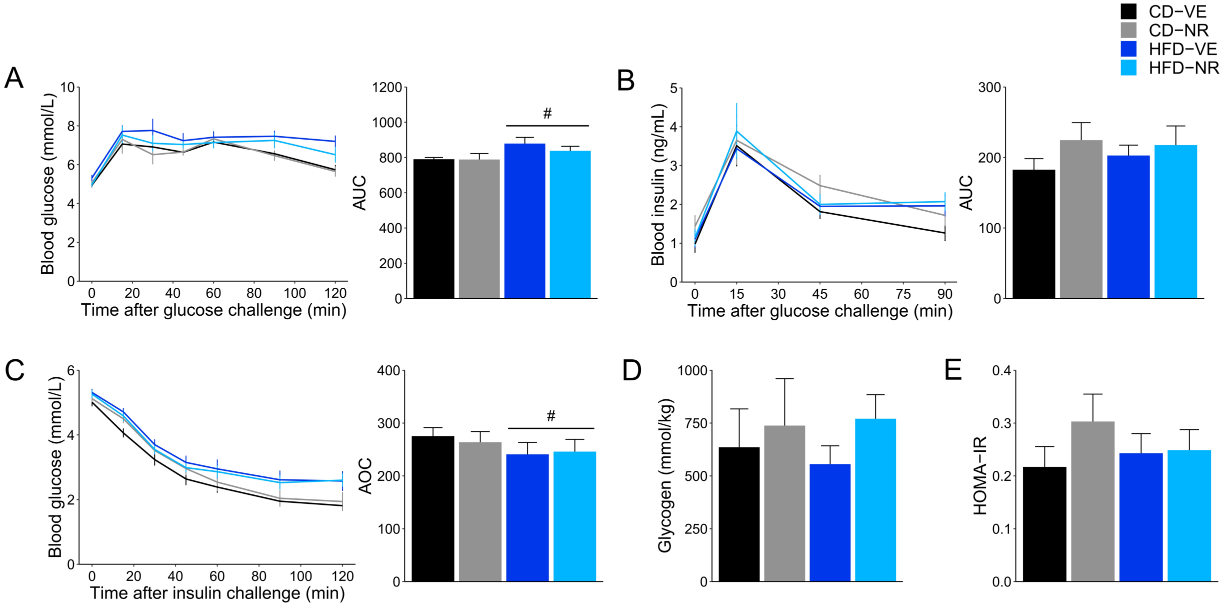

Supplementary Figure 2: Glucose and insulin tolerance tests

(A) Oral glucose tolerance test (OGTT). Blood glucose over 120 minutes after glucose challenge and area under the curve (AUC). Diet effect: # $p < 0.05$.

(B) Insulin secretion (OGTT). Blood insulin levels after glucose challenge and area under the curve (AUC).

(C) Insulin tolerance test (ITT). Blood glucose over 120 minutes after insulin injection and area over the curve (AOC). Diet effect: # $p < 0.05$.

(D) Average of hepatic glycogen levels.

(E) Homeostatic Model Assessment of Insulin Resistance

Data are shown as mean ± SEM. n = 6-8.

Supplementary Figure 3

A

	CD		HFD	
	VE	NR	VE	NR
CA	8/8 (100 %)	6/6 (100 %)	8/8 (100 %)	8/8 (100 %)
DCA	8/8 (100 %)	6/6 (100 %)	8/8 (100 %)	8/8 (100 %)
LCA	8/8 (100 %)	6/6 (100 %)	8/8 (100 %)	8/8 (100 %)
α-MCA	8/8 (100 %)	6/6 (100 %)	8/8 (100 %)	8/8 (100 %)
β-MCA	8/8 (100 %)	6/6 (100 %)	8/8 (100 %)	8/8 (100 %)
UDCA	8/8 (100 %)	6/6 (100 %)	8/8 (100 %)	8/8 (100 %)
TLCA	4/8 (50 %)	2/6 (33 %)	4/8 (50 %)	6/8 (75 %)
β-TMCA	3/8 (38 %)	2/6 (33 %)	2/8 (25 %)	5/8 (62 %)
γ-MCA	2/8 (25 %)	1/6 (17 %)	3/8 (38 %)	2/8 (25 %)
TCA	1/8 (12 %)	0/6 (0 %)	1/8 (12 %)	3/8 (38 %)
TCDCA	1/8 (12 %)	0/6 (0 %)	1/8 (12 %)	1/8 (12 %)
TDCA	0/8 (0 %)	0/6 (0 %)	0/8 (0 %)	3/8 (38 %)
α-TMCA	0/8 (0 %)	1/6 (17 %)	0/8 (0 %)	0/8 (0 %)
GCA	0/8 (0 %)	0/6 (0 %)	0/8 (0 %)	0/8 (0 %)
GCDCA	0/8 (0 %)	0/6 (0 %)	0/8 (0 %)	0/8 (0 %)
GDCA	0/8 (0 %)	0/6 (0 %)	0/8 (0 %)	0/8 (0 %)
GUDCA	0/8 (0 %)	0/6 (0 %)	0/8 (0 %)	0/8 (0 %)
γ-TMCA	0/8 (0 %)	0/6 (0 %)	0/8 (0 %)	0/8 (0 %)
TUDCA	0/8 (0 %)	0/6 (0 %)	0/8 (0 %)	0/8 (0 %)

B

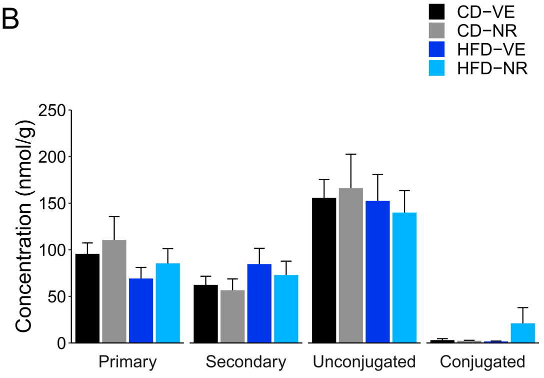

C

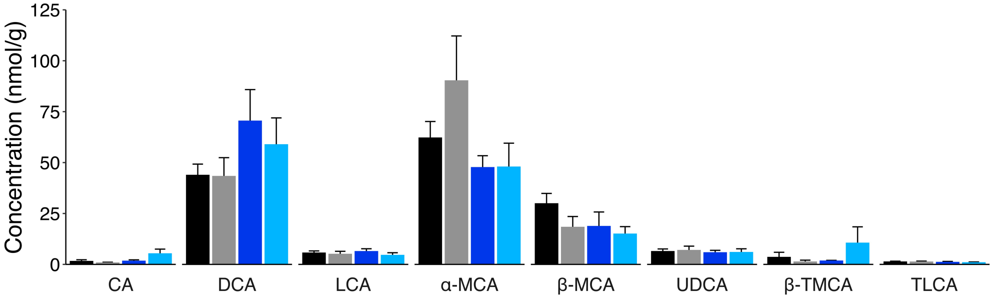

Supplementary Figure 3: Targeted analysis of cecal bile acids by LC-MS/MS
(A) List of all target bile acids set in our LC-MS methodology and the percentage of bile acids quantificatied in each diet and treatment groups.
(B) Average of all identified primary, secondary, unconjugated and conjugated bile acids.
(C) Average of all identified bile acids in (A).
Data are shown as mean ± SEM. n = 6-8.

Cholic acid (CA); Chenodeoxycholic acid (CDCA); Ddeoxycholic acid (DCA); Lithocholic acid (LCA); α-Muricholic acid (α-MCA); β-Muricholic acid (β-MCA); Ursodeoxycholic acid (UDCA); Taurolithocholic acid (TLCA; Tauro-β-muricholic acid (β-TMCA) γ-Muricholic Acid (γ-MCA); Taurocholic acid (TCA); Taurochenodeoxycholic acid (TCDCA); Taurodeoxycholic acid (TDCA); α-Tauromuricholic acid (TMCA); Glycocholic acid (GCA); Glycochenodeoxycholic acid (GCDCA); Glycodeoxycholic acid (GDCA); Glycoursodeoxycholic acid (GUDCA); γ-Tauromuricholic acid (γ-TMCA); Tauroursodeoxycholic acid (TUDCA).

Supplementary Figure 4

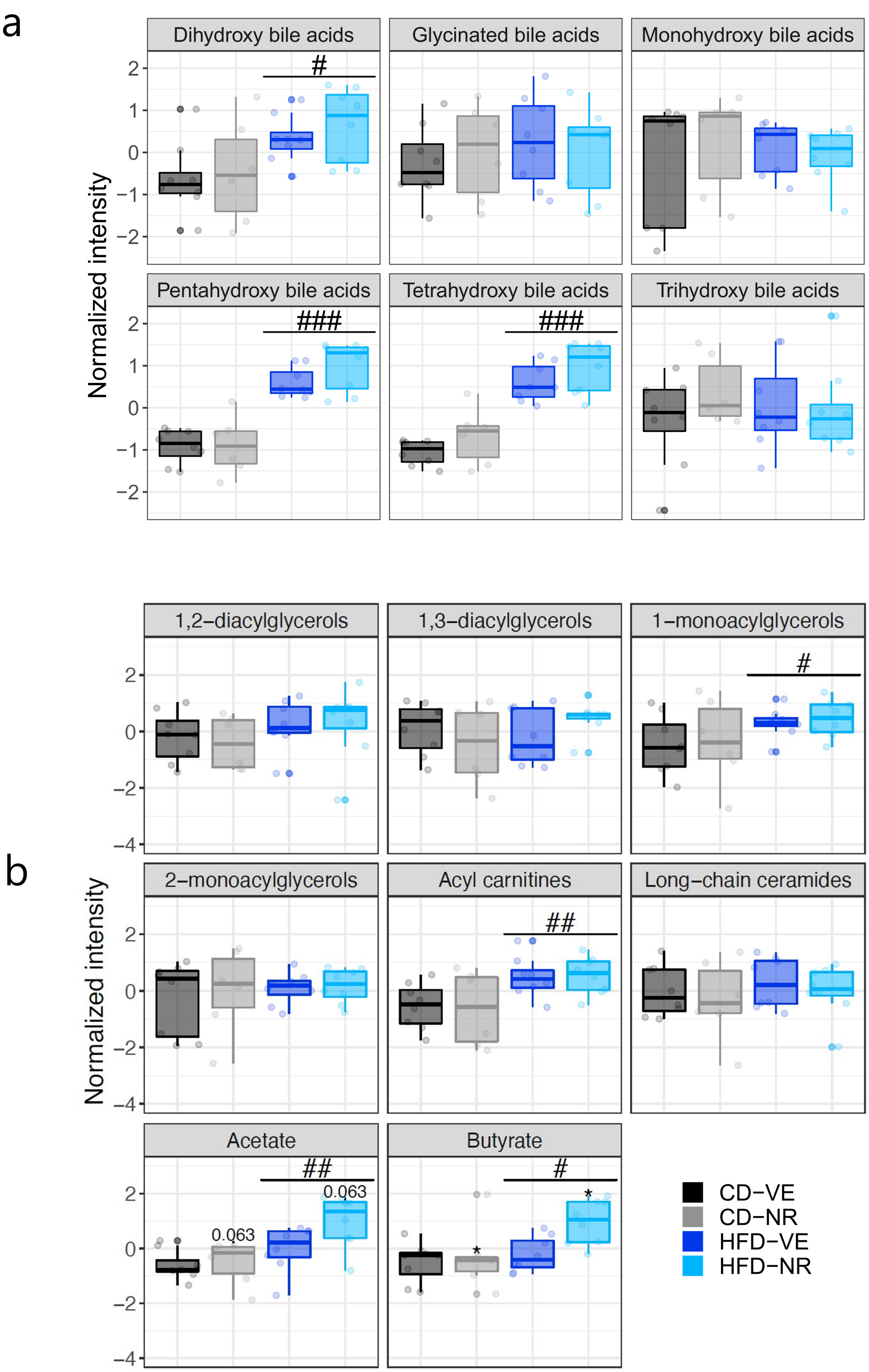

Supplementary Figure 4: Untargeted analysis of plasma metabolites by LC-MS/MS

(a) Average of different classes of identified bile acids. Diet effect: # $p < 0.05$; ### $p < 0.001$.
(b) Average of diacylglycerols (DAGs), monoacylglycerols (MAGs), acylcarnitines, ceramides and the short-chain fatty acids acetate and butyrate. Diet effect: # $p < 0.05$; ## $p < 0.01$¨. NR effect: * $p < 0.05$. Two-way ANOVA was applied in all cases. Boxplots are depicted using the minimum and the maximum shown by the whiskers and the 1st, 2nd, and 3rd quartiles. n = 6-8.

Supplementary Figure 5

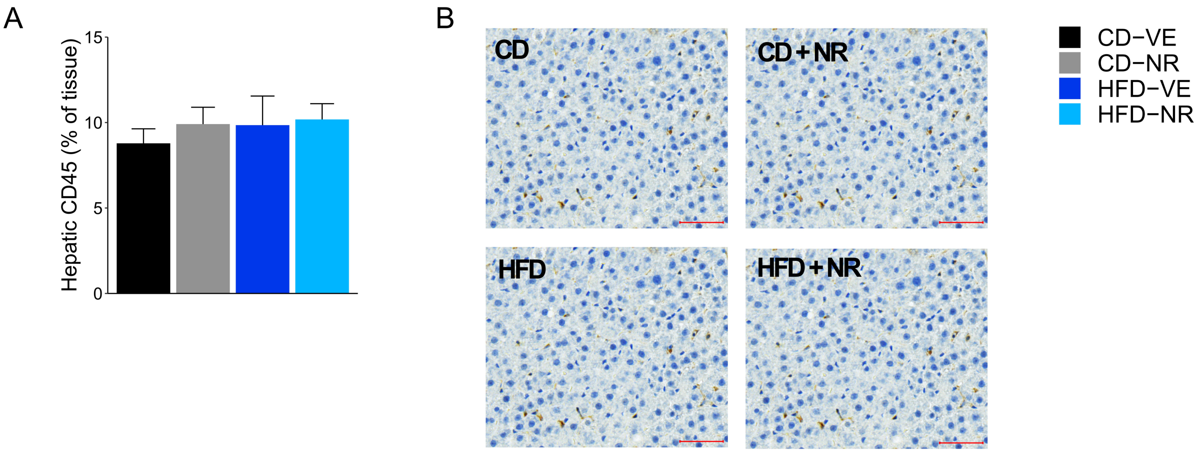

Supplementary Figure 5: Liver CD45 protein quantification

(A) Relative abundances of liver CD45 and (B) representative tissue immunostaining. Scale bar: 50 µm. Data are shown as mean ± SEM. n = 6-8.

Supplementary Figure 6

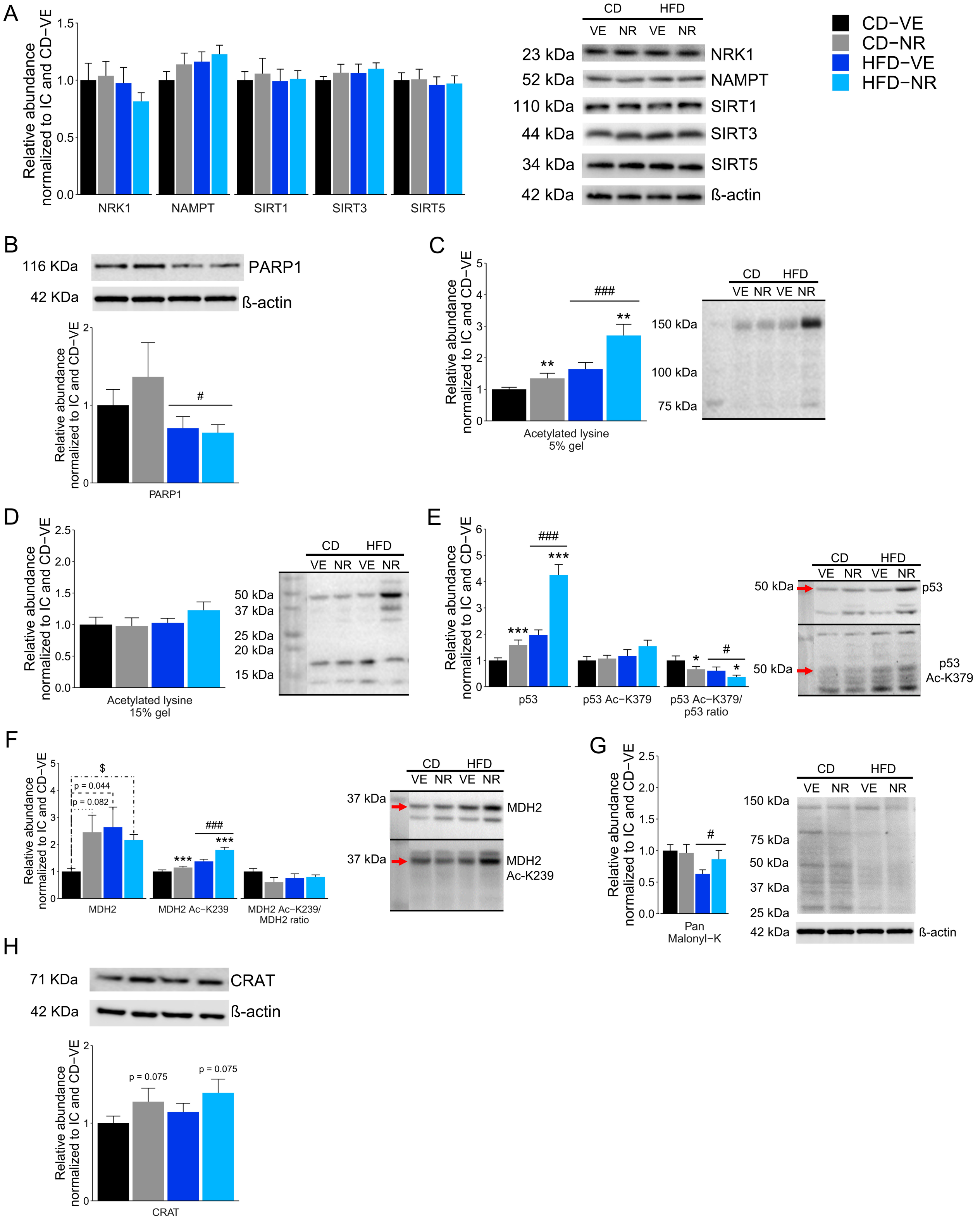

Supplementary Figure 6: Liver protein abundance of NAD^+-related enzymes

(A) Relative abundances of NRK1, NAMPT, SIRT1, SIRT3 and SIRT5.
(B) Relative abundances of liver PARP1 and representative bands from western blots. Multiple comparison tests: Diet effect # $p < 0.05$. Values are normalized to internal control (IC) and CD-VE means.
(C) Relative abundance of acetylated lysine residues in 5 and (D)15% polyacrylamide gel and representative bands from western blots. Main treatment effect:. Multiple comparison tests: Diet effect ### $p < 0.001$.
NR effect ** $p < 0.01$.
(E) Relative abundances of p53, p53 Ac-K379, and p53 Ac-K379 to p53 ratio and representative bands from western blots. Diet effect: # $p < 0.05$; : ### $p < 0.001$. NR effect: * $p < 0.05$; *** $p < 0.001$,
(F) Relative abundances of MDH2, MDH2 Ac-K239, and MDH2 Ac-K239 to MDH2 ratio and representative bands from western blots. Diet effect: ### $p < 0.001$. NR effect: *** $p < 0.001$. Interaction: $ $p < 0.05$.
(G) Relative abundances and representative bands of pan-malonyl-lysine western blots. Multiple comparison tests: Diet effect # $p < 0.05$.
H) Relative abundances of liver carnitine acetyltransferase (CRAT) and representative bands from western blots.
Values are normalized to internal control (IC) and CD-VE means.

Data are shown as mean ± SEM. n = 6-8.

Supplementary Figure 7

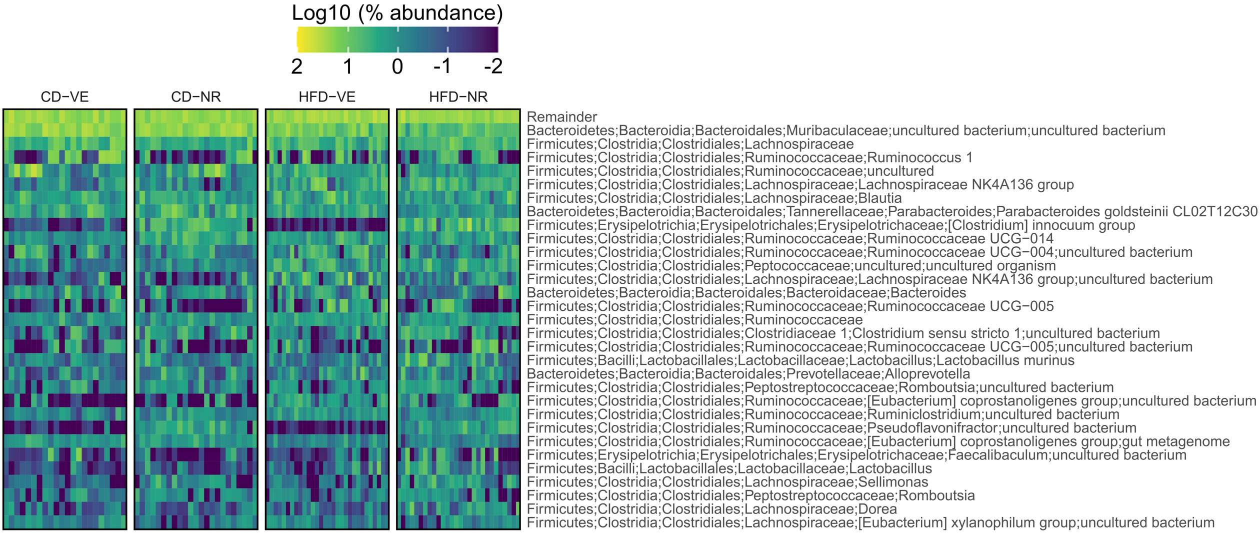

Supplementary Figure 7: Abundance heatmaps of the 30 most abundant species on average
Data are shown based on diet and treatment combined and species are sorted by the overall mean abundance.

Supplementary Figure 8

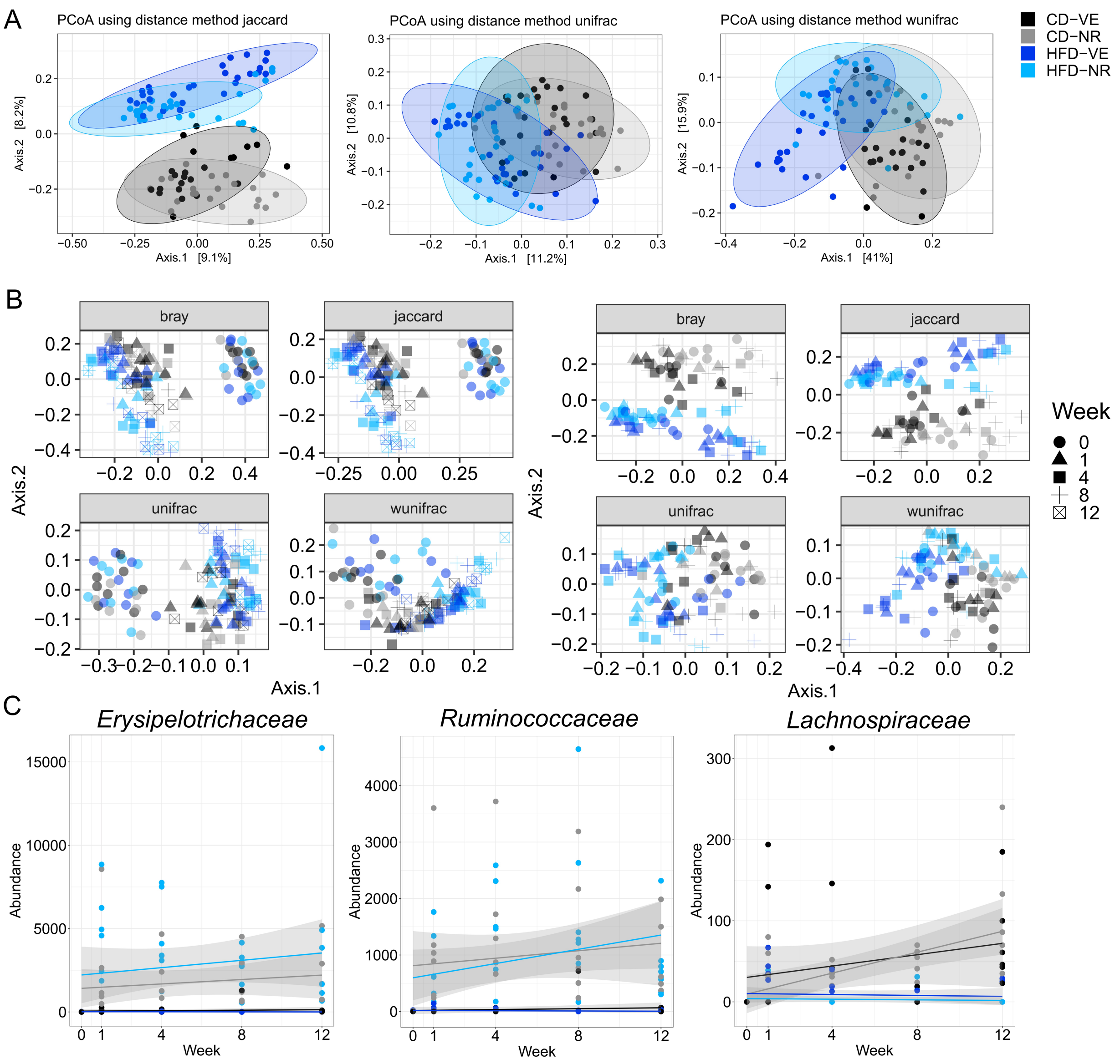

Supplementary Figure 8: Effect of NR on the rat microbiome diversity

(A) PCoA plots of ß-diversity based on Jaccard, unweighted and weighted UniFrac distance matrices, respectively. Colors are according to diet and treatment groups. Axes numbers indicate the percentage of variation explained.

(B) β-diversity calculated based on Bray-Curtis, Jaccard, unweighted UniFrac and weighted UniFrac distance matrices before and after week 0 exclusion. n = 2-8.

(C) Linear mixed effect (LME) models for regression analyses from week 0 to 12 confirming that the abundance of *Ruminococcaceae* and *Erysipelotrichaceae* species was significantly impacted by NR treatment. *Lachnospiraceae* species was significantly impacted by diet.

Supplementary Figure 9

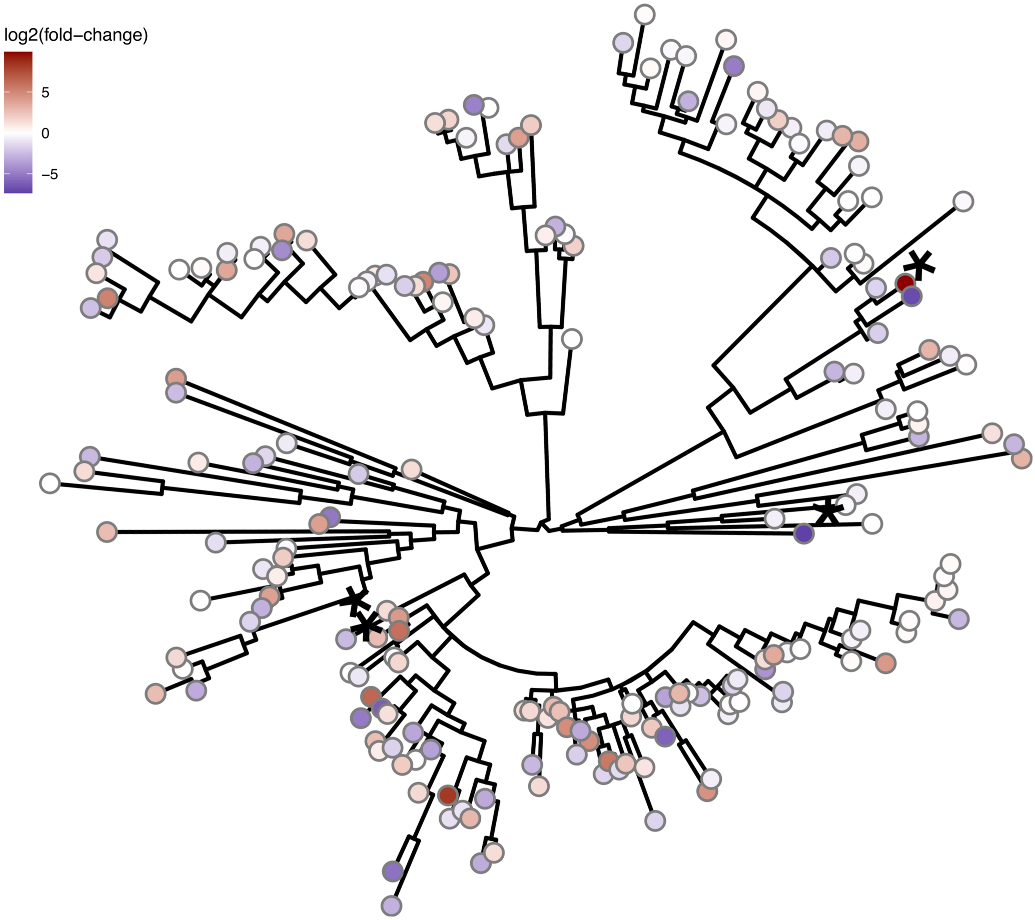

Supplementary Figure 9: Effect of NR on the mouse microbiome
Phylogenetic tree displaying fold-change in the abundance of features between control and NR-treated mice. Significant changes are marked with *.

Supplementary Figure 10

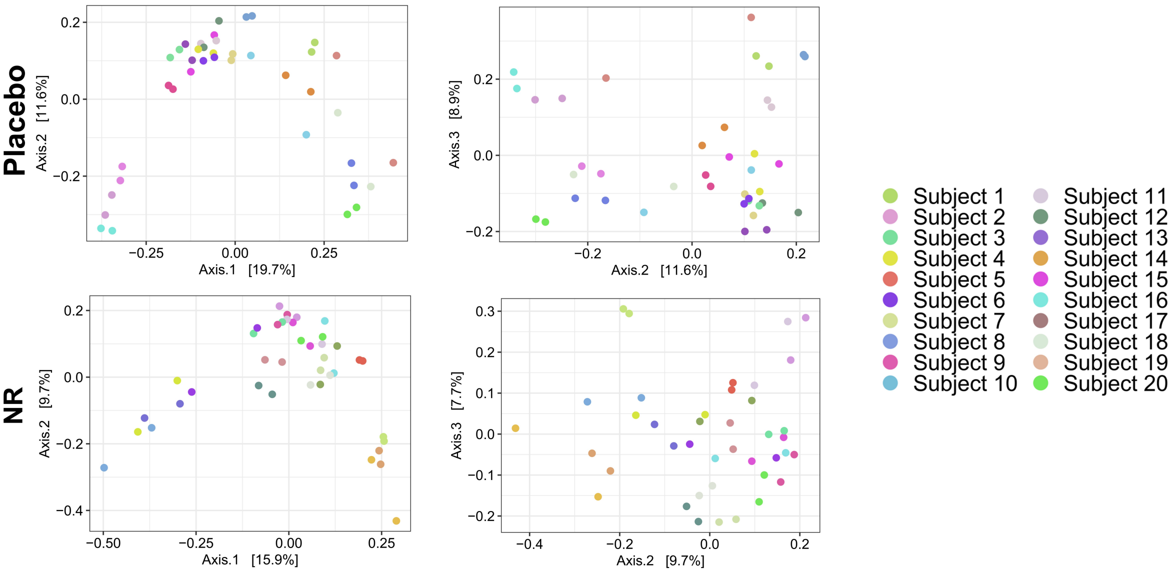

Supplementary Figure 10: PCoA plots based on Bray-Curtis distance matrix.
Samples are colored based on patients. Samples from the same patient are relatively closer to each other in both placebo and NR intervention cohorts. n = 20 per group.

Supplementary Figure 11

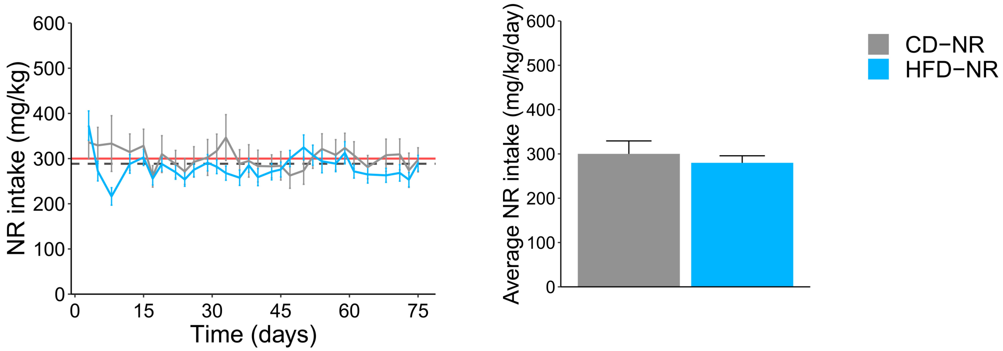

Supplementary Figure 11: NR intake per cage over 11 weeks of study
Single measurements (left) and the average of the whole period (right). The red line marks the aim of 330 mg/kg/day (10% spill included) and the dashed line marks average intake for both diet groups over the whole period. Shown as mean ± SEM. n = 4 (2 animals per cage).

Supplementary Figure 12

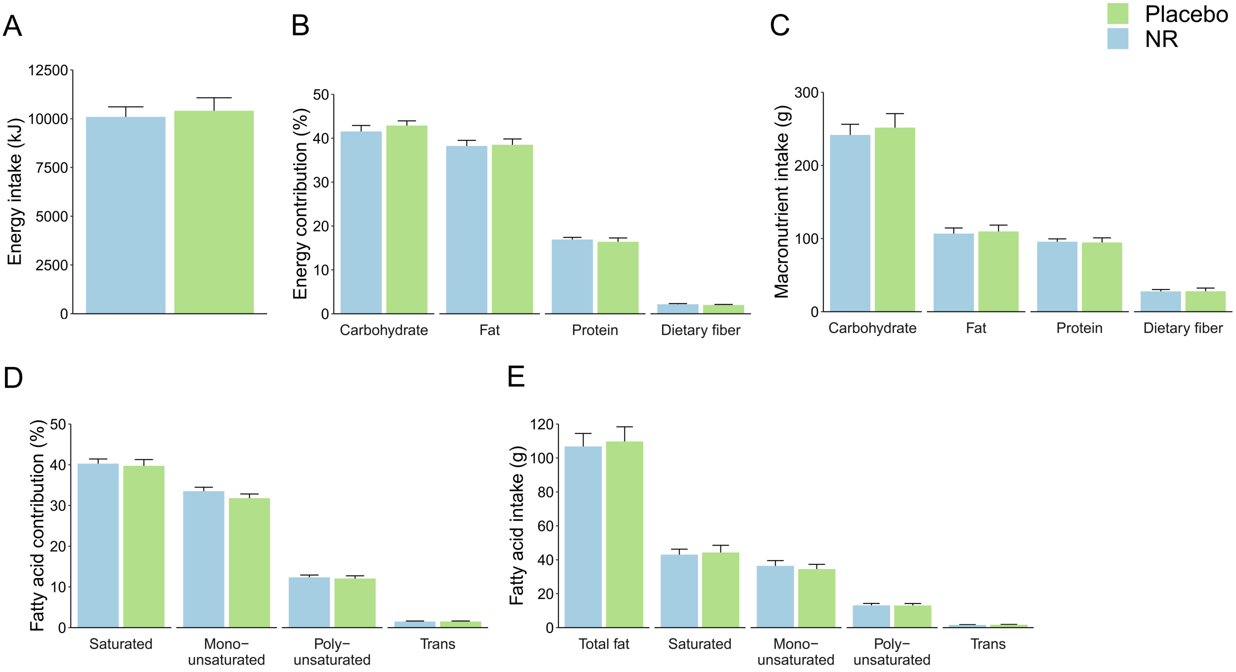

Supplementary Figure 12: Dietary intake in study participants assessed three days before placebo or NR supplementation was initiated

A. Total energy intake (kJ). B. Energy distribution derived from macronutrient sources represented in percentage of total energy (%). C. Distribution of macronutrients (g). D. Distribution of fatty acids (%). E. Total fat intake (g) and the distribution of fatty acids (g). Student t-tests and Mann-Whitney Rank Sum Tests were applied where appropriate. Data are shown as mean + SEM (n=20 per treatment group). No statistically significant differences were found between treatment groups.

Supplementary Table 1: Plasma bile acids profile

Bile acid levels were determined using untargeted LC-MS in plasma. n = 6-8

CD- Control diet, HFD- High-fat diet, NR- Nicotinamide riboside, VE- Vehicle.

**Metabolite**	**Diet group**	**Fold change**	**p-value**
Taurodeoxycholic acid (TDCA)	CD	2.3	0.016
	HFD	2.1	0.038
Glycocholic acid (GCA)	CD	2.5	0.049
	HFD	-	NS

Supplementary Table 2

Fecal Metagenomics: Bray Curtis

	Permanova						
Diet	Group 1	Group 2	Sample size	Permutations	pseudo-F	p-value	q-value
	CD	HFD	101	999	13,61487724	0,001	0,001

Treatment	Group 1	Group 2	Sample size	Permutations	pseudo-F	p-value	q-value
	NR	VE	101	999	6,503521992	0,001	0,001

	Group 1	Group 2	Sample size	Permutations	pseudo-F	p-value	q-value
Diet + Treatment	CD+NR	CD+VE	43	999	3,003112338	0,001	0,001
	CD+NR	HFD+NR	49	999	8,064302926	0,001	0,001
	CD+NR	HFD+VE	51	999	9,230415934	0,001	0,001
	CD+VE	HFD+NR	50	999	12,34245015	0,001	0,001
	CD+VE	HFD+VE	52	999	8,604928492	0,001	0,001
	HFD+NR	HFD+VE	58	999	6,44571728	0,001	0,001

	Permdisp						
Diet	Group 1	Group 2	Sample size	Permutations	F-value	p-value	q-value
	CD	HFD	101	1999	3,955424688	0,0495	0,0495

Treatment	Group 1	Group 2	Sample size	Permutations	F-value	p-value	q-value
	NR	VE	101	999	10,01541859	0,001	0,001

	Group 1	Group 2	Sample size	Permutations	F-value	p-value	q-value
Diet + Treatment	CD+NR	CD+VE	43	999	2,579007767	0,091	0,182
	CD+NR	HFD+NR	49	999	1,271713104	0,235	0,3312
	CD+NR	HFD+VE	51	999	4,783262611	0,032	0,182
	CD+VE	HFD+NR	50	999	1,026944028	0,276	0,3312
	CD+VE	HFD+VE	52	999	0,265598209	0,618	0,618
	HFD+NR	HFD+VE	58	999	2,579169763	0,088	0,182

Fecal Metagenomics: Unweighted UniFrac

	Permanova						
Diet	Group 1	Group 2	Sample size	Permutations	pseudo-F	p-value	q-value
	CD	HFD	101	999	8,501964869	0,001	0,001

Treatment	Group 1	Group 2	Sample size	Permutations	pseudo-F	p-value	q-value
	NR	VE	101	999	3,319111471	0,001	0,001

	Group 1	Group 2	Sample size	Permutations	pseudo-F	p-value	q-value
Diet + Treatment	CD+NR	CD+VE	43	999	2,213025801	0,002	0,002
	CD+NR	HFD+NR	49	999	4,534235314	0,001	0,0012
	CD+NR	HFD+VE	51	999	6,047002137	0,001	0,0012
	CD+VE	HFD+NR	50	999	6,194012881	0,001	0,0012
	CD+VE	HFD+VE	52	999	6,73924135	0,001	0,0012
	HFD+NR	HFD+VE	58	999	3,977640063	0,001	0,0012

	Permdisp						
Diet	Group 1	Group 2	Sample size	Permutations	F-value	p-value	q-value
	CD	HFD	101	999	12,46659969	0,001	0,001

Treatment	Group 1	Group 2	Sample size	Permutations	F-value	p-value	q-value
	NR	VE	101	999	3,001671419	0,094	0,094

	Group 1	Group 2	Sample size	Permutations	F-value	p-value	q-value
Diet + Treatment	CD+NR	CD+VE	43	999	2,593415292	0,093	0,186
	CD+NR	HFD+NR	49	999	1,891178624	0,184	0,2208
	CD+NR	HFD+VE	51	999	2,24215626	0,145	0,2175
	CD+VE	HFD+NR	50	999	9,334425238	0,005	0,015
	CD+VE	HFD+VE	52	999	14,34993241	0,003	0,015
	HFD+NR	HFD+VE	58	999	0,008536495	0,925	0,925

Fecal Metagenomics: Weighted UniFrac

	Permanova						
Diet	Group 1	Group 2	Sample size	Permutations	pseudo-F	p-value	q-value
	CD	HFD	101	999	23,85188549	0,001	0,001

Treatment	Group 1	Group 2	Sample size	Permutations	pseudo-F	p-value	q-value
	NR	VE	101	999	7,192995458	0,001	0,001

	Group 1	Group 2	Sample size	Permutations	pseudo-F	p-value	q-value
Diet + Treatment	CD+NR	CD+VE	43	999	2,483644898	0,033	0,033
	CD+NR	HFD+NR	49	999	18,28265203	0,001	0,0012
	CD+NR	HFD+VE	51	999	13,30262318	0,001	0,0012

	Group 1	Group 2	Sample size	Permutations	pseudo-F	p-value	q-value
	CD+VE	HFD+NR	50	999	20,76254155	0,001	0,0012
	CD+VE	HFD+VE	52	999	10,3512035	0,001	0,0012
	HFD+NR	HFD+VE	58	999	9,220130543	0,001	0,0012

	**Permdisp**						
**Diet**	**Group 1**	**Group 2**	**Sample size**	**Permutations**	**F-value**	**p-value**	**q-value**
	CD	HFD	101	999	1,000921912	0,312	0,312

**Treatment**	**Group 1**	**Group 2**	**Sample size**	**Permutations**	**F-value**	**p-value**	**q-value**
	NR	VE	101	999	0,265298406	0,596	0,596

	**Group 1**	**Group 2**	**Sample size**	**Permutations**	**F-value**	**p-value**	**q-value**
**Diet + Treatment**	CD+NR	CD+VE	43	999	1,559943765	0,216	0,432
	CD+NR	HFD+NR	49	999	0,01180825	0,917	0,917
	CD+NR	HFD+VE	51	999	0,18496158	0,639	0,8184
	CD+VE	HFD+NR	50	999	2,521063846	0,112	0,336
	CD+VE	HFD+VE	52	999	3,152899758	0,079	0,336
	HFD+NR	HFD+VE	58	999	0,166470981	0,682	0,8184

Fecal Metagenomics: Jaccard

	**Permanova**						
**Diet**	**Group 1**	**Group 2**	**Sample size**	**Permutations**	**pseudo-F**	**p-value**	**q-value**
	CD	HFD	101	999	7,331999569	0,001	0,001

**Treatment**	Group 1	Group 2	Sample size	Permutations	pseudo-F	p-value	q-value
	NR	VE	101	999	3,653344993	0,001	0,001

	**Group 1**	**Group 2**	**Sample size**	**Permutations**	**pseudo-F**	**p-value**	**q-value**
**Diet + Treatment**	CD+NR	CD+VE	43	999	2,135175081	0,002	0,002
	CD+NR	HFD+NR	49	999	4,825112553	0,001	0,0012
	CD+NR	HFD+VE	51	999	5,251084241	0,001	0,0012
	CD+VE	HFD+NR	50	999	6,047017562	0,001	0,0012
	CD+VE	HFD+VE	52	999	5,434329319	0,001	0,0012
	HFD+NR	HFD+VE	58	999	4,48309805	0,001	0,0012

	**Permdisp**						
**Diet**	**Group 1**	**Group 2**	**Sample size**	**Permutations**	**F-value**	**p-value**	**q-value**
	CD	HFD	101	999	2,736519124	0,103	0,103

Treatment	Group 1	Group 2	Sample size	Permutations	F-value	p-value	q-value
	NR	VE	101	999	5,700790248	0,016	0,016

	**Group 1**	**Group 2**	**Sample size**	**Permutations**	**F-value**	**p-value**	**q-value**
**Diet + Treatment**	CD+NR	CD+VE	43	999	2,423953823	0,102	0,204
	CD+NR	HFD+NR	49	999	1,01890818	0,314	0,3768
	CD+NR	HFD+VE	51	999	0,000505219	0,983	0,983
	CD+VE	HFD+NR	50	999	7,487234041	0,005	0,03
	CD+VE	HFD+VE	52	999	5,390646302	0,025	0,075
	HFD+NR	HFD+VE	58	999	1,805928447	0,157	0,2355

Jejunum Metagenomics:

	**Permanova**						
**Diet**	**Group 1**	**Group 2**	**Sample size**	**Permutations**	**pseudo-F**	**p-value**	**q-value**
	CD	HFD	23	999	2,043871078	0,096	0,096

**Treatment**	**Group 1**	**Group 2**	**Sample size**	**Permutations**	**pseudo-F**	**p-value**	**q-value**
	NR	VE	23	999	1,582748657	0,178	0,178

	**Group 1**	**Group 2**	**Sample size**	**Permutations**	**pseudo-F**	**p-value**	**q-value**
**Diet + Treatment**	CD+NR	CD+VE	10	999	1,347400671	0,344	0,672
	CD+NR	HFD+NR	9	999	0,371857692	0,884	0,884
	CD+NR	HFD+VE	10	999	0,928217215	0,451	0,672
	CD+VE	HFD+NR	13	999	2,245808559	0,103	0,309
	CD+VE	HFD+VE	14	999	2,200975559	0,092	0,309
	HFD+NR	HFD+VE	13	999	0,74021492	0,56	0,672

Ileum Metagenomics:

	**Permanova**						
**Diet**	**Group 1**	**Group 2**	**Sample size**	**Permutations**	**pseudo-F**	**p-value**	**q-value**
	CD	HFD	30	999	3,012305499	0,034	0,034

**Treatment**	**Group 1**	**Group 2**	**Sample size**	**Permutations**	**pseudo-F**	**p-value**	**q-value**
	NR	VE	30	999	1,509700058	0,178	0,178

	**Group 1**	**Group 2**	**Sample size**	**Permutations**	**pseudo-F**	**p-value**	**q-value**
**Diet + Treatment**	CD+NR	CD+VE	14	999	1,048884198	0,316	0,367
	CD+NR	HFD+NR	14	999	1,763739283	0,135	0,29

	CD+NR	HFD+VE	14	999	3,635989226	0,049	0,29
	CD+VE	HFD+NR	16	999	1,357094093	0,261	0,367
	CD+VE	HFD+VE	16	999	1,78282197	0,145	0,29
	HFD+NR	HFD+VE	16	999	1,087798749	0,367	0,367

	**Permdisp**						
**Diet**	**Group 1**	**Group 2**	**Sample size**	**Permutations**	**F-value**	**p-value**	**q-value**
	CD	HFD	30	999	0,79345641	0,375	0,375

Cecum Metagenomics:

	**Permanova**						
**Diet**	**Group 1**	**Group 2**	**Sample size**	**Permutations**	**pseudo-F**	**p-value**	**q-value**
	CD	HFD	30	999	5,004255204	0,001	0,001

**Treatment**	**Group 1**	**Group 2**	**Sample size**	**Permutations**	**pseudo-F**	**p-value**	**q-value**
	NR	VE	30	999	1,65236786	0,095	0,095

	**Group 1**	**Group 2**	**Sample size**	**Permutations**	**pseudo-F**	**p-value**	**q-value**
**Diet + Treatment**	CD+NR	CD+VE	14	999	0,916236171	0,469	0,469
	CD+NR	HFD+NR	14	999	2,608841626	0,017	0,034
	CD+NR	HFD+VE	14	999	2,175898033	0,048	0,072
	CD+VE	HFD+NR	16	999	4,613138235	0,001	0,006
	CD+VE	HFD+VE	16	999	3,837870323	0,002	0,006
	HFD+NR	HFD+VE	16	999	2,242230199	0,061	0,0732

	**Permdisp**						
**Diet**	**Group 1**	**Group 2**	**Sample size**	**Permutations**	**F-value**	**p-value**	**q-value**
	CD	HFD	30	999	0,164947435	0,568	0,568

**Treatment**

	**Group 1**	**Group 2**	**Sample size**	**Permutations**	**F-value**	**p-value**	**q-value**
**Diet + Treatment**	CD+NR	CD+VE	14	999	0,033201853	0,789	0,933
	CD+NR	HFD+NR	14	999	0,004506866	0,917	0,933
	CD+NR	HFD+VE	14	999	0,295124091	0,482	0,933
	CD+VE	HFD+NR	16	999	0,003468484	0,933	0,933
	CD+VE	HFD+VE	16	999	0,199933245	0,493	0,933
	HFD+NR	HFD+VE	16	999	0,153441345	0,52	0,933

Proximal Colon Metagenomics:

	Permanova						
**Diet**	**Group 1**	**Group 2**	**Sample size**	**Permutations**	**pseudo-F**	**p-value**	**q-value**
	CD	HFD	29	999	5,172665502	0,001	0,001

**Treatment**	**Group 1**	**Group 2**	**Sample size**	**Permutations**	**pseudo-F**	**p-value**	**q-value**
	NR	VE	29	999	1,404331283	0,182	0,182

	**Group 1**	**Group 2**	**Sample size**	**Permutations**	**pseudo-F**	**p-value**	**q-value**
**Diet + Treatment**	CD+NR	CD+VE	14	999	0,854929247	0,54	0,54
	CD+NR	HFD+NR	13	999	2,05670535	0,033	0,0495
	CD+NR	HFD+VE	14	999	2,871625002	0,002	0,004
	CD+VE	HFD+NR	15	999	3,723244794	0,002	0,004
	CD+VE	HFD+VE	16	999	4,084175362	0,001	0,004
	HFD+NR	HFD+VE	15	999	1,480863047	0,143	0,1716

	Permdisp						
**Diet**	**Group 1**	**Group 2**	**Sample size**	**Permutations**	**F-value**	**p-value**	**q-value**
	CD	HFD	29	999	1,778440435	0,11	0,11

**Treatment**

	**Group 1**	**Group 2**	**Sample size**	**Permutations**	**F-value**	**p-value**	**q-value**
**Diet + Treatment**	CD+NR	CD+VE	14	999	0,007235718	0,929	0,929
	CD+NR	HFD+NR	13	999	3,386553815	0,002	0,006
	CD+NR	HFD+VE	14	999	0,231605541	0,543	0,6516
	CD+VE	HFD+NR	15	999	4,947345009	0,002	0,006
	CD+VE	HFD+VE	16	999	0,228745369	0,441	0,6516
	HFD+NR	HFD+VE	15	999	7,78614991	0,006	0,012

Distal Colon Metagenomics:

	Permanova						
**Diet**	**Group 1**	**Group 2**	**Sample size**	**Permutations**	**pseudo-F**	**p-value**	**q-value**
	CD	HFD	30	999	5,510595476	0,001	0,001

**Treatment**	**Group 1**	**Group 2**	**Sample size**	**Permutations**	**pseudo-F**	**p-value**	**q-value**
	NR	VE	30	999	2,066682807	0,027	0,027

	Group 1	Group 2	Sample size	Permutations	pseudo-F	p-value	q-value
**Diet + Treatment**	CD+NR	CD+VE	14	999	1,298183246	0,225	0,225
	CD+NR	HFD+NR	14	999	3,508597039	0,003	0,006
	CD+NR	HFD+VE	14	999	2,96717337	0,008	0,012
	CD+VE	HFD+NR	16	999	5,152408125	0,001	0,003
	CD+VE	HFD+VE	16	999	3,980481077	0,001	0,003
	HFD+NR	HFD+VE	16	999	2,740210546	0,012	0,0144

	**Permdisp**						
**Diet**	**Group 1**	**Group 2**	**Sample size**	**Permutations**	**F-value**	**p-value**	**q-value**
	CD	HFD	30	999	2,813168814	0,034	0,034

**Treatment**	Group 1	Group 2	Sample size	Permutations	F-value	p-value	q-value
	NR	VE	30	999	0,059897686	0,741	0,741

	Group 1	Group 2	Sample size	Permutations	F-value	p-value	q-value
**Diet + Treatment**	CD+NR	CD+VE	14	999	0,012278275	0,893	0,941
	CD+NR	HFD+NR	14	999	0,257011552	0,43	0,941
	CD+NR	HFD+VE	14	999	0,215752467	0,516	0,941
	CD+VE	HFD+NR	16	999	0,101039646	0,618	0,941
	CD+VE	HFD+VE	16	999	0,015756547	0,819	0,941
	HFD+NR	HFD+VE	16	999	0,0011406	0,941	0,941

Supplementary Table 3: Multiple reaction monitoring transitions of the analytes used for LC-MS of the NAD^+ metabolome.

Name	MRM transitions	Cone voltage (CV)	Collision energy(CE)
Nam	123 > 80	2	17
NA	124 > 80	2	17
MeNam	137 > 80	25	20
Me2PY/Me4PY	153 > 110	20	17
NR	255 > 123	25	13
NAR	256 > 124	24	11
NMN	335 > 123	25	14
NAMN	336 > 124	24	12
ADPR	560 > 136	30	30
NADH	667 > 136	25	40
NAD	665 > 136	25	45
NAAD	666 > 136	25	45
NADPH	747 > 136	25	45
$NADP^+$	745 > 136	25	50
NAADP	746 > 136	25	45
Nam d4	127 > 84	2	18
NA d4	128 > 84	2	18
MeNam d4	141 > 80	25	20
NADH d4	670 > 136	25	43
NAD d4	668 > 136	25	43
TRP d5	210 > 192	10	9

Supplementary Table 4

16S rRNA Amplicon primers

Name	Sequence 5'-3
FW.A501	AATGATACGGCGACCACCGAGATCTACACATCGTACGTATGGTAATTGTGTGCCAGCMGCCGCGGTAA
FW.A502	AATGATACGGCGACCACCGAGATCTACACACTATCTGTATGGTAATTGTGTGCCAGCMGCCGCGGTAA
FW.A503	AATGATACGGCGACCACCGAGATCTACACTAGCGAGTTATGGTAATTGTGTGCCAGCMGCCGCGGTAA
FW.A504	AATGATACGGCGACCACCGAGATCTACACCTGCGTGTTATGGTAATTGTGTGCCAGCMGCCGCGGTAA
FW.A505	AATGATACGGCGACCACCGAGATCTACACTCATCGAGTATGGTAATTGTGTGCCAGCMGCCGCGGTAA
FW.A506	AATGATACGGCGACCACCGAGATCTACACCGTGAGTGTATGGTAATTGTGTGCCAGCMGCCGCGGTAA
FW.A507	AATGATACGGCGACCACCGAGATCTACACGGATATCTTATGGTAATTGTGTGCCAGCMGCCGCGGTAA
FW.A508	AATGATACGGCGACCACCGAGATCTACACGACACCGTTATGGTAATTGTGTGCCAGCMGCCGCGGTAA
FW.B501	AATGATACGGCGACCACCGAGATCTACACCTACTATATATGGTAATTGTGTGCCAGCMGCCGCGGTAA
FW.B502	AATGATACGGCGACCACCGAGATCTACACCGTTACTATATGGTAATTGTGTGCCAGCMGCCGCGGTAA
FW.B503	AATGATACGGCGACCACCGAGATCTACACAGAGTCACTATGGTAATTGTGTGCCAGCMGCCGCGGTAA
FW.B504	AATGATACGGCGACCACCGAGATCTACACTACGAGACTATGGTAATTGTGTGCCAGCMGCCGCGGTAA
FW.B505	AATGATACGGCGACCACCGAGATCTACACACGTCTCGTATGGTAATTGTGTGCCAGCMGCCGCGGTAA
FW.B506	AATGATACGGCGACCACCGAGATCTACACTCGACGAGTATGGTAATTGTGTGCCAGCMGCCGCGGTAA
FW.B507	AATGATACGGCGACCACCGAGATCTACACGATCGTGTTATGGTAATTGTGTGCCAGCMGCCGCGGTAA
FW.B508	AATGATACGGCGACCACCGAGATCTACACGTCAGATATATGGTAATTGTGTGCCAGCMGCCGCGGTAA
RE.A701	CAAGCAGAAGACGGCATACGAGATAACTCTCGAGTCAGTCAGCCGGACTACHVGGGTWTCTAAT
RE.A702	CAAGCAGAAGACGGCATACGAGATACTATGTCAGTCAGTCAGCCGGACTACHVGGGTWTCTAAT
RE.A703	CAAGCAGAAGACGGCATACGAGATAGTAGCGTAGTCAGTCAGCCGGACTACHVGGGTWTCTAAT
RE.A704	CAAGCAGAAGACGGCATACGAGATCAGTGAGTAGTCAGTCAGCCGGACTACHVGGGTWTCTAAT
RE.A705	CAAGCAGAAGACGGCATACGAGATCGTACTCAAGTCAGTCAGCCGGACTACHVGGGTWTCTAAT
RE.A706	CAAGCAGAAGACGGCATACGAGATCTACGCAGAGTCAGTCAGCCGGACTACHVGGGTWTCTAAT
RE.A707	CAAGCAGAAGACGGCATACGAGATGGAGACTAAGTCAGTCAGCCGGACTACHVGGGTWTCTAAT
RE.A708	CAAGCAGAAGACGGCATACGAGATGTCGCTCGAGTCAGTCAGCCGGACTACHVGGGTWTCTAAT
RE.A709	CAAGCAGAAGACGGCATACGAGATGTCGTAGTAGTCAGTCAGCCGGACTACHVGGGTWTCTAAT
RE.A710	CAAGCAGAAGACGGCATACGAGATTAGCAGACAGTCAGTCAGCCGGACTACHVGGGTWTCTAAT
RE.A711	CAAGCAGAAGACGGCATACGAGATTCATAGACAGTCAGTCAGCCGGACTACHVGGGTWTCTAAT
RE.A712	CAAGCAGAAGACGGCATACGAGATTCGCTATAAGTCAGTCAGCCGGACTACHVGGGTWTCTAAT
RE.B701	CAAGCAGAAGACGGCATACGAGATAAGTCGAGAGTCAGTCAGCCGGACTACHVGGGTWTCTAAT
RE.B702	CAAGCAGAAGACGGCATACGAGATATACTTCGAGTCAGTCAGCCGGACTACHVGGGTWTCTAAT
RE.B703	CAAGCAGAAGACGGCATACGAGATAGCTGCTAAGTCAGTCAGCCGGACTACHVGGGTWTCTAAT
RE.B704	CAAGCAGAAGACGGCATACGAGATCATAGAGAAGTCAGTCAGCCGGACTACHVGGGTWTCTAAT
RE.B705	CAAGCAGAAGACGGCATACGAGATCGTAGATCAGTCAGTCAGCCGGACTACHVGGGTWTCTAAT
RE.B706	CAAGCAGAAGACGGCATACGAGATCTCGTTACAGTCAGTCAGCCGGACTACHVGGGTWTCTAAT
RE.B707	CAAGCAGAAGACGGCATACGAGATGCGCACGTAGTCAGTCAGCCGGACTACHVGGGTWTCTAAT
RE.B708	CAAGCAGAAGACGGCATACGAGATGGTACTATAGTCAGTCAGCCGGACTACHVGGGTWTCTAAT
RE.B709	CAAGCAGAAGACGGCATACGAGATGTATACGCAGTCAGTCAGCCGGACTACHVGGGTWTCTAAT
RE.B710	CAAGCAGAAGACGGCATACGAGATTACGAGCAAGTCAGTCAGCCGGACTACHVGGGTWTCTAAT
RE.B711	CAAGCAGAAGACGGCATACGAGATTCAGCGTTAGTCAGTCAGCCGGACTACHVGGGTWTCTAAT
RE.B712	CAAGCAGAAGACGGCATACGAGATTCGCTACGAGTCAGTCAGCCGGACTACHVGGGTWTCTAAT
Index primer	ATTAGAWACCCBDGTAGTCCGGCTGACTGACT
Read 1 Seq	ATTGTGTGCCAGCMGCCGCGGTAA
Read 2 Seq	CAGCCGGACTACHVGGGTWTCTAAT
